# Hnrnpk is essential for embryonic limb bud development as a transcription activator and a collaborator of insulator protein Ctcf

**DOI:** 10.1038/s41418-023-01207-z

**Published:** 2023-08-22

**Authors:** Yuyu Chen, Taifeng Zhou, Zhiheng Liao, Wenjie Gao, Jinna Wu, Shun Zhang, Yongyong Li, Hengyu Liu, Hang Zhou, Caixia Xu, Peiqiang Su

**Affiliations:** 1 Department of Spine Surgery, Guangdong Provincial Key Laboratory of Orthopedics and Traumatology, the First Affiliated Hospital of Sun Yat-sen University, Guangzhou, 510080 China; 2 Department of Orthopaedics, Sun Yat-sen Memorial Hospital of Sun Yat-sen University, Guangzhou, 510120 China; 3 Department of Breast Surgery, Affiliated Cancer Hospital & Institute of Guangzhou Medical University, Guangzhou, 510095 China; 4 Precision Medicine Institute, the First Affiliated Hospital of Sun Yat-sen University, Guangzhou, 510080 China; 5 Research Center for Translational Medicine, the First Affiliated Hospital of Sun Yat-sen University, Guangzhou, 510080 China

**Keywords:** Development, Gene regulation

## Abstract

Proper development of the limb bud relies on the concordance of various signals, but its molecular mechanisms have not yet been fully illustrated. Here we report that heterogeneous nuclear ribonucleoprotein K (hnRNPK) is essential for limb bud development. Its ablation in the limb bud results in limbless forelimbs and severe deformities of the hindlimbs. In terms of mechanism, hnRNPK functions as a transcription activator for the vital genes involved in the three regulatory axes of limb bud development. Simultaneously, for the first time we elucidate that hnRNPK binds to and coordinates with the insulator protein CCCTC binding factor (CTCF) to maintain a three-dimensional chromatin architecture. Ablation of hnRNPK weakens the binding strength of CTCF to topologically associating domain (TAD) boundaries, then leading to the loose TADs, and decreased interactions between promoters and enhancers, and further decreased transcription of developmental genes. Our study establishes a fundamental and novel role of hnRNPK in regulating limb bud development.

## Introduction

The limb of the vertebrae originates from the limb bud and is controlled by a complicated regulatory network. The limb bud emerges around embryonic day (E) 26 in humans and E9.5 in mice, composing mesenchymal cells from the lateral plate mesoderm. The limb bud develops along three axes—the proximal-distal axis (PD axis), anterior-posterior axis (AP axis), and dorsal-ventral axis (DV axis)—eventually forming an intact and well-organized limb. The false pattern of pivotal signals results in musculoskeletal deformities [1]. The PD axis, running from shoulders to fingers, is essential for the proper elongation and segmentation of the limb bud, and it is predominantly guided by the apical ectodermal ridge (AER) region located at the ectoderm covering the tip of the limb bud [2]. Cells in AER secrete fibroblast growth factor 8 (FGF8), which is triggered by FGF10 from limb bud mesenchyme, and FGF8 prompts FGF10 secretion via a positive regulatory loop [3]. The AP axis, running from digits I to V, is specified by the zone of polarizing area (ZPA) expressing sonic hedgehog (SHH) [4], and its disorder results in abnormal digit formation, such as polydactyly or oligodactyly [5]. The DV axis is arranged from the back of the hand to the palm, and is regulated by LIM homeobox transcription factor 1 beta (LMX1B) and WNT7A signals [6].

The three axes during embryonic limb development require complex transcriptional regulation [7]. For instance, the transcription of *SHH* is a complicated process due to the long distance (>1 Mb) between its promoter and enhancer. The prominent enhancer of *SHH* is located in the intron 5 of limb development membrane protein 1 (*LMBR1*), known as the ZPA regulatory sequence (*ZRS*), and interacts with the *SHH* promoter through a long-range chromatin loop [8, 9]. A similar mechanism is involved in the transcription of homeobox (*HOX*) family genes. The *HOX* family consists of four clusters, with *HOXA* and *HOXD* clusters playing vital roles in limb development. There is a dynamic switch in the expression pattern of the *HOXD* cluster, including *HOXD9*-*HOXD13*, whose regulatory elements are bimodal and flanked by two topologically associating domains (TADs) to ensure the region- and time- specific pattern of the *HOXD* cluster during embryonic limb development [10–13].

Three-dimensional chromatin structures contain compartments, TADs, and loops, in which chromatin is highly folded and self-interacted [14, 15]. TAD is one kind of three-dimensional genome structure that is separated by boundaries consisting of insulators [16], and the pivotal insulator protein of TAD formation is the zinc-finger transcription factor CCCTC binding factor (CTCF). CTCF cooperates with the multi-subunit protein complex Cohesin to shape functional boundaries [17]. Deleting or inverting CTCF binding sites results in the false formation of TAD, leading to a gene misexpression pattern. Removal of CTCF binding sites at E12.5 mouse limb bud disturbs the expression of *Sox9* and its adjacent gene *Kcnj2* [18], indicating that CTCF affects developmental gene expression. The deletion or inhibition of CTCF and Cohesin reportedly leads to alterations of intra- and inter-TADs, which also leads to gene misexpression [19–21], especially during the development of the brain and kidney [22, 23]. During limb bud development, the disturbance of CTCF binding and TAD shaping results in a moderate decrease of the gene expression of *SHH* and *HOXD* [24, 25]. However, the regulatory factors of CTCF in limb bud development are poorly understood.

hnRNPK is an RNA-binding protein (RBP) according to its structure [26], but it possesses the capacity to bind to DNA, RNA, and protein [27, 28]. As a multifunctional protein, hnRNPK participates in important physiological and pathological processes through transcriptional, post-transcriptional, and chromatin remodeling mechanisms [29, 30]. A *de-novo* loss-of-function mutation in human gene *HNRNPK* is reportedly related to Au-Kline syndrome (AKS, OMIM: 616580) [31], characterized by remarkable deformities in the limb and spine. Recently, hnRNPK was shown to promote the osteogenesis of mesenchymal stem cells (MSCs) by interacting with lncRNA-OG [32], and it also regulated osteoclastogenesis through glycogen synthase kinase-3β (GSK3β) [33]. We previously reported that a novel mutation in the *ZRS* resulted in preaxial polydactyly (PPD) type I by inducing ectopic expression of *SHH*, and we further identified hnRNPK as a mediator that enhanced the interactions between mutant *ZRS* and the *SHH* promoter in vitro [34]. Nonetheless, no in vivo experimental evidence has been reported till now to elucidate the regulatory role of hnRNPK in the limb bud development.

In this study, we investigated the function of Hnrnpk in limb bud development by specifically deleting *Hnrnpk* in mouse limb buds, and its ablation resulted in limbless forelimbs and severe deformities of hindlimbs. In terms of mechanism, we illustrated that Hnrnpk regulated the transcription of key genes involved in the three regulatory axes in limb bud development as a transcription activator. Additionally, we discovered that the ablation of Hnrnpk resulted in the global remodeling of chromatin architecture, especially TADs. Our high-throughput assays demonstrated that Hnrnpk possessed the capacity to interact with the insulator protein Ctcf to ensure proper TAD formation, which guaranteed the normal interaction between regulatory elements of genes during the development of the limb bud.

## Materials and methods

### Mice

**Fig. 1:**
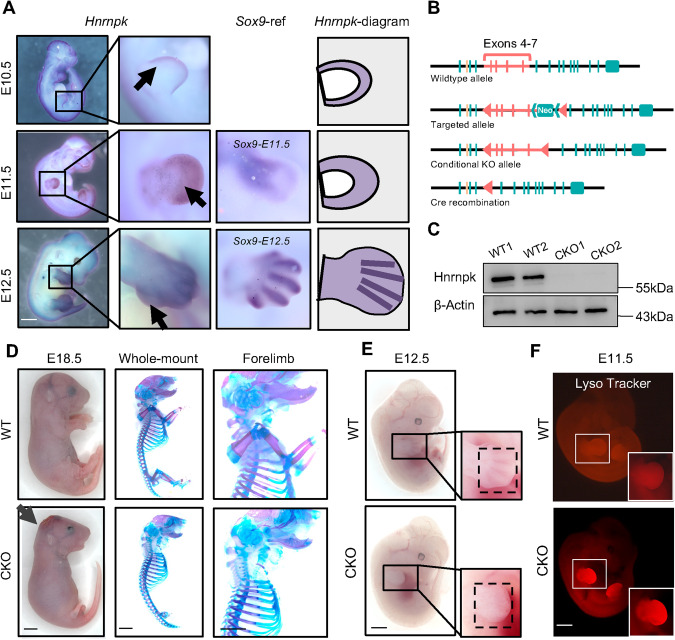
*Hnrnpk* is expressed in developing limb buds and is essential for proper limb bud formation. **A** Representative WISH images showed *Hnrnpk* (left panel) and *Sox9* (middle panel) expression at E10.5 (top), E11.5 (middle), and E12.5 (bottom) limb buds. The *Hnrnpk*-diagram (right panel) presented the range of *Hnrnpk* signals. The black arrows indicated stronger signals detected in the periphery and cartilage primordia region of the limb buds. Scale bar: 1 mm. **B** Construction protocol of *floxed-Hnrnpk* mice. Exons 4–7 of the *Hnrnpk* gene was flanked by *loxP* sites. The horizontal lines indicated chromatin, vertical lines indicated the exons of *Hnrnpk*, yellow line represented the exon of adjacent gene *Rmi1*, and triangles indicated *loxP* sites inserted. **C** The efficient knockout of Hnrnpk in limb buds was confirmed by WB using proteins from E11.5 WT and CKO limb buds. β-Actin was used as the loading control. **D** Representative images of general observation (left panel) and skeletal preparation (right panel) of E18.5 WT and CKO embryos. The black arrow indicated the hematoma on the E18.5 CKO head. Scale bar: 1 mm. **E** Representative images of general observation of E12.5 WT and CKO embryos. The dotted boxes showed regions of mesenchyme condensation. Scale bar: 1 mm. **F** Representative Lyso tracker images of E11.5 WT and CKO limb buds. The white boxes indicated the magnification of the forelimb. Scale bar: 1 mm.

All animal experiments were approved by Institutional Animal Care and Use Committee, Sun Yat-sen University (SYSU-IACUC-2021-000758), and the mice were kept in the Laboratory Animal Center of Sun Yat-sen University. *Floxed-Hnrnpk* mice carried a couple of *loxP* sites flanking exons 4–7 of the *Hnrnpk* gene were generated by Cyagen Biosciences. A schematic diagram was shown in Fig. 1B. Briefly, the exons 4–7 were selected as the conditional knock-out region. We engineered the target vector containing Neo cassette flanked by Rox sites and the knock-out region flanked by loxP sites. And the constitutive knockout allele was obtained after Cre-mediated recombination. *Prrx1-Cre* mice were purchased from the Jackson Laboratory (stock #005584). *Floxed-Trp53* mice were a kindly gift from Prof. Jin Liu. The *Rosa26-tdTomato* mice were purchased from Jackson Laboratory (stock #007914). We used both male and female embryos for analysis, as sex could not be clearly identified. Genotyping was conducted with the primers (*Floxed-Hnrnpk* primer forward: GTCTCTCGCTCTGTCTTTGTGGC; reverse: GGAAGGGCTCAGATTAAGTGGCAA) following the PCR reaction conditions (initial denaturation at 94 °C for 3 min, denaturation at 94 °C for 30 s, annealing at 60 °C for 30 s, extension at 72 °C for 30 s, repetition of 35 cycles, and additional extension at 72 °C for 2 min). Three or more littermate groups were examined, and representative images were shown.

### Whole-mount in situ hybridization (WISH)

WISH experiments were performed as previously described, using digoxygenin-labeled (Roche, 11277073910) antisense RNA probes, and sense RNA probes as negative control [35]. The probes of *Col2a1*, *Sox9*, *Lmx1b*, *Fgf10*, and *Hnrnpk* were transcribed from the cDNA of WT limb bud cells. To ensure the exact same developmental stages, the embryos used in the WISH experiments for each time point or each probe came from the same pregnant mice. The probe sequences were described in Table S2. The probes of *Shh* and *Fgf8* were transcribed from corresponding plasmids from Addgene (13999 and 22088).

### Primary limb bud cell culture

The isolation and culture of limb bud cell was previously reported [34]. E11.5 WT or CKO embryos were isolated, and their amniotic membrane were removed for genotyping using dissecting scissors and forceps under a stereoscopic microscope (Leica M205FA). The limb bud tissue was cut down and put into the sterile PBS and washed. Then, the limb bud tissue was digested with 0.25% trypsin for 5 min at 37 °C water baths. After centrifuged, the cell mass was resuspended and the cells were seeded and cultured at 37 °C with 5% CO_2_ in complete medium (low-glucose DMEM with L-glutamine supplemented with 1% penicillin-streptomycin solution and 10% fetal bovine serum).

### qPCR

Total RNA was extracted using TRIZOL reagent (Invitrogen), and 1000 ng of total RNA was used to synthesize cDNA with a Prime-Script RT reagent kit (TaKaRa) according to the manufacturer’s protocol. qPCR was performed on a Light Cycler 480 Real-time PCR system (Roche Light Cycler 480) using TB Green Premix Taq II (TaKaRa) and corresponding primers. Relative expression levels were calculated by the 2^−ΔΔCt^ method and *Actb* served as internal control for normalization. The primers used in qPCR were shown in Table S1.

### Immunoblotting analysis

Western blotting was performed according to standard procedures. The following antibodies were used: anti-hnRNPK (Abcam 39975, 1:5000), anti-p53 (CST 2527, 1:1000), anti-phospho-p53 (CST 9284, 1:500), anti-p21 (Abcam 109520, 1:1000), anti-Bax (CST 14796, 1:1000), anti-Bcl2 (CST 3498, 1:1000), anti-CTCF (Millipore 07-729, 1:2000), anti-H3K4me1 (Active Motif 61634, 1:1000), anti-H3K4me3 (CST 9751, 1:1000), anti-H3K27ac (Active Motif 39134, 1:1000), anti-RNA polymerase II (Santa-cruz sc-56767,1:1000), anti-β-ACTIN (Affinity AF7018, 1:2000), goat anti-rabbit IgG H&L (Abcam ab205718, 1:2000), and goat anti-mouse IgG H&L (Abcam ab205719, 1:2000). The quantification of WB was exerted using software imageJ (v 1.51). The original pictures of WB were shown in Supplementary materials Western blot.

### Skeletal preparation

The skeletal preparation was carried out as previously described [36], and then was photographed under a stereoscopic microscope (Zeiss Axio Zoom v16) in glycerol.

### Lysotracker staining

E11.5 WT and CKO embryos were stripped from the uterus and extra-embryonic membranes were removed. The embryos were then incubated in Lyso tracker (Thermo Fisher Scientific) staining solution (150 nM Lyso tracker in Hanks balanced salt solution) at 37 °C for 45 min. The embryos were fixed in 4% paraformaldehyde overnight and exposed under a stereoscopic microscope with fluorescence (Zeiss Axio Zoom v16) to detect apoptotic signals.

### Cell counting kit-8 assay (CCK-8)

Primary limb bud cells were seeded at a density of 1 × 10^4^ per well in 96-well plates and cultured for 0,1,2,3,4,5, and 6 days. CCK-8 solution (Glpbio) was added to each well and incubated for 3 h, followed by measurement of the absorbance at 450 nm using a microplate reader (TECAN sunrise).

### Flow cytometric analysis

Flow cytometric analysis assessments and data analysis were conducted using CytoFLEX (Beckman Coulter) flow cytometers and FlowJo10.8.1 software. The following antibodies were used: CD140B-PE (Biolegend 136009), CD90-PE (Biolegend 105201), and CD82-FITC (Biolegend 342102).

### Micromass culture system

*Hnrnpk*^*fl/fl*^ limb bud cells were seeded at the density of 2 × 10^7^ cells/ml. The high-density droplets were placed carefully in the center of each well in a 24-well plate. The cells were allowed to adhere at 37 °C for 1.5 h, followed by the infection of adenovirus with *GFP* or *Cre* (Shanghai Genechem). The cells were induced with chondrogenic medium (DMEM/F12 medium containing 10% fetal bovine serum and 50 μg/ml ascorbic acid). The medium was changed every other day and the staining was performed on day 6 and day 12.

### Co-immunoprecipitation and DNase/RNase digestion

Protein was extracted using a lysis buffer containing the 1% phosphatase and protease inhibitor. The protein was incubated sequentially with primary antibodies and Protein G manganic beads (Invitrogen), and the beads were then incubated in wash buffer containing DNase I (Invitrogen) or RNase A (Thermo Fisher Scientific) for 1.5 h on ice. Finally, the protein was eluted by a sample buffer and analyzed by western blotting. The following antibodies were used: anti-hnRNPK (Bethyl A300-676A-M, 1:200) and anti-CTCF (Millipore 07-729, 1:200).

### Electron microscopy imaging

E11.5 WT and CKO limb bud were isolated and fixed with 2.5% glutaraldehyde and dehydrated with gradient ethanol. The tissues were then embedded and cut into slices, stained with uranyl acetate for 15 min and lead citrate for 5 min. The slices were imaged under electron microscopy (JEM-1200EX). Three biological replicates were observed, and 4–5 cells were observed in each replicate.

### RNA-seq

Total RNA was extracted from E11.5 WT and CKO limb bud cells and the libraries were constructed using the VAHTS Stranded mRNA-seq Library Prep Kit for Illumina v2. The libraries’ quality was checked using Qubit 2100, and the concentrations were determined from the analysis profiles. Genes expression with a change fold ≥2 or ≤ −2, *p* value < 0.05 were identified as differentially expressed genes, and the GO enrichment analysis and KEGG pathway analysis were performed using DAVID [37].

### ATAC-seq

ATAC-seq was performed as previously described [38]. WT and CKO limb bud cells were harvested, and about 5 × 10^5^ cells were pretreated with DNase to remove free-floating DNA and DNA of dead cells. After centrifugation, the cells were resuspended in cold lysis buffer, followed by incubation on ice for 3 min. After lysis, 1 ml of ATAC-seq RSB was added and centrifuged. After centrifugation, nuclei were resuspended in a transposition mix containing 5 × TD buffer and Tn5 transposase. Samples were incubated for 30 min at 37 °C with shaking for transposition reactions. Reactions were cleaned up with Zymo DNA Clean and Concentrator 5 columns. DNA was resuspended in 2×HiFi PCR mix with Nextera i5 primer (N5xx) and Nextera i7 primer (N7xx) for PCR reaction. After PCR, size selection was performed with EpiTM DNA Clean Beads (Epibiotek R1809), and the supernatant was transferred to a new tube containing fresh beads to capture fragments ranging from 250 to 350 bp. Libraries were quantified with a Bioptic Qsep100 Analyzer (Bioptic Inc.) and paired-end sequenced with read lengths of 150 bp.

### ATAC-seq analysis

ATAC-seq data analysis was performed as previously reported [39]. The reads were trimmed to remove the adapter sequences and aligned to the mouse genome (mm10) using Bowtie2 [40]. Peak-calling was performed using MACS2 [41] software in ATAC-seq mode to exclude the PCR duplicates and mitochondrial reads. The ATAC-seq signal was presented using Integrative Genomics Viewer (IGV) software [42]. Peak comparison, annotation, and motif enrichment were performed using ChIPSeeker [43] and HOMER [44]. GO enrichment analysis and KEGG pathway analysis were performed using DAVID [37].

### CRISPRi/sgRNA plasmid construction

The pLV hU6-sgRNA-hUbC-dCas9-KRAB-T2a-Puro plasmid was available from Addgene (71236). Targeted sgRNAs were inserted into the plasmid using BsmBI sites. The sequences of sgRNAs were as described in Table S3.

### Vectors transduction and lentivirus/adenovirus infection

Lentivirus infection was performed according to the manufacturer’s protocol of the GeneCopoeia Lenti-Pac kit. Briefly, HEK293T cells were plated in high glucose DMEM (Gibco) with 10% FBS and 1% penicillin-streptomycin. The cells were co-transfected with 2.5 µg dCas9 plasmid and packing plasmids (pLP1, pLP2 and pLP-vsvg) using Lipofectamine 3000 (Thermo Fisher Scientific). After 8–14 h, the transfection medium was changed and the medium with lentivirus was harvested after 48 h. The lentiviral supernatant was filtered through 0.45 µm filters to eliminate residual cells and cell fragments. After the cells were attached to the dish with 70–80% confluency, the adenovirus or lentivirus was added to the non-FBS medium (10^9^ PFU/ml). The same volume medium with 10% FBS was added to the cells, and the medium was changed after 24 h. The cells were cultured for another 24 h before the next step experiments.

### Immunofluorescent staining

The limb bud tissue was isolated and fixed with 4% paraformaldehyde, dehydrated using 30% sucrose solution, and cut into frozen sections. The slides were blocked with 5% bovine serum albumin, then incubated with anti-Ki67 (CST 9192, 1:250) or anti-Lmx1b (Proteintech 18278-1, 1:500) overnight and then the species-matched Alexa Fluor 555 for 3 h. After staining with DAPI, the slides were observed under a microscope (Olympus BX63). As for TdT-mediated dUTP nick end labeling (TUNEL) assay was conducted according to the manufacturer’s instructions for the one-step TUNEL Apoptosis Assay Kit (Beyotime). Briefly, the sections of limb bud tissue was incubation with 20 μg/ml proteinase K for 30 min at 37 °C, then the TUNEL detective mixture was pipetted onto the sections, followed by incubation at 37 °C for 60 min in darkness. After staining with DAPI, the slides were observed under a microscope (Olympus BX63)

E11.5 WT limb bud cells were seeded on cover slides and fixed with 4% paraformaldehyde, followed by permeabilization with 0.5% Triton X-100. The cover slips were blocked with 5% bovine serum albumin and then incubated overnight with anti-hnRNPK (Abcam ab52600, 1:250). The cells were incubated in a secondary antibody (CST 4413, 1:1000) for 3 h. After washing with PBS, the cover slips were incubated with anti-CTCF (Millipore 07-729, 1:500) or anti-RNA polymerase II (Santa-cruz sc-56767, 1:500) overnight again and with anti-rabbit secondary antibody (Invitrogen A-21206, 1:1000) or anti-mouse secondary antibody (Invitrogen A-11001, 1:1000) for 1 h. After staining with DAPI, the cover slips were observed under a laser scanning confocal microscope (Zeiss LSM 880).

### CUT&RUN-seq

Procedure and analysis of CUT&RUN-seq was performed as previously described [45]. WT and CKO limb bud cells were harvested, counted, and centrifuged. Aliquots containing 5 × 10^5^ cells were washed and resuspended in wash buffer. Activated concanavalin A-coated magnetic beads were added to the cells, and the setup was incubated at room temperature for 15 min. The unbound supernatant was removed, and the bead-bound cells were resuspended in Dig-wash Buffer containing primary antibodies on a rotating platform overnight. Primary antibodies used were anti-hnRNPK (Bethyl A300-676A-M, 1:100), anti-CTCF (Sigma-Aldrich 07-729, 1:100), anti-H3K4me3 (Sigma-Aldrich 04-745, 1:100), and H3K4me1 (Active Motif 39635, 1:100). The cells were washed to remove unbound primary antibodies. PA/G-MNase were added and placed on the tube rotator at 4 °C for 1 h. The cells were then washed to remove unbound PA/G-MNase and resuspended in Dig-wash Buffer in wet ice to chill down. After chilling down, 100 mM of CaCl2 was added to the suspension with a gentle vortex and rotated at 4 °C for 30 min. After rotation, 2× STOP Buffer was added and incubation was continued for 30 min at 37 °C to release CUT&RUN fragments from the insoluble nuclear chromatin. For sample normalization with NG-seq, 12.5 pg of Sample Normalization Spike-in DNA (CST 40366) was added to each reaction. The supernatant containing digested chromatin was transferred to a fresh tube, and the DNA was extracted with Phenol Chloroform Extraction following the manufacturer’s protocol. The extracted DNA was processed for library generation using the QIAseq Ultralow Input Library Kit (QIAGEN), following the manufacturer’s protocol. Libraries were quantified with a Bioptic Qsep100 Analyzer (Bioptic Inc.) and paired-end sequenced with read lengths of 150 bp. After quality control, the reads were trimmed to remove the adapter sequences and aligned to the mouse genome (mm10) using Bowtie2 [40] and Spike-in was used for normalization. Peak-calling was performed using MACS2 [41] software to exclude the PCR duplicates and mitochondrial reads. Peak comparison, annotation, and motif enrichment were performed using ChIPSeeker [43] and MEME-chip [46]. GO enrichment analysis and KEGG pathway analysis were performed using DAVID [37].

### Hi-C assay

Hi-C assay was performed as previously reported [15]. About 5 × 10^5^ limb bud cells were collected from each embryo. To decrease the intra-group difference, four littermates of same genotype for each group were mixed and about 2 × 10^6^ cells were used for the Hi-C assay. Cells were cross-linked with 1% formaldehyde and then quenched with 0.125 M glycine. Cross-linked cells were lysed in lysis buffer, followed by incubation at 37 °C for 15 min. Chromatin Capture Beads were added and incubated for 10 min at room temperature. Chromatin was digested with the Hi-C restriction enzyme and the fragments were marked with a biotinylated nucleotide. The chromatin was then ligated for 4 h at 16 °C, followed by elution in reverse cross-linking buffer at 65 °C for 3 h. The DNA was treated with RNase A and proteinase K at 37 °C for 30 min and purified with 1.8× EpiTM DNA Clean Beads Ligation. The DNA was suspended in digestion buffer and subjected to enzymatic shearing to shear chromatin fragments to an average size between 200 bp and 500 bp by incubating the tube at 30 °C for 15 min. The fragments were processed for library generation using the QIAseq Ultralow Input Library Kit (QIAGEN), following the manufacturer’s protocol, and the biotinylated ligation junctions were captured with streptavidin beads after the adapter ligation. The libraries were quantified with a Bioptic Qsep100 Analyzer (Bioptic Inc.) and paired-end sequenced. After sequencing, 3.3 × 10^3^ Mbp were acquired in the WT group and 3.1 × 10^3^ Mbp in the CKO group.

### Hi-C analysis

Capture Hi-C processing FASTQ files (paired-end Illumina data) were processed with HiC-Pro [47] using Bowtie2 [40] to map short reads to the reference genome (mm10). After the mapping, filtering and deduplication steps were performed using the HiC-Pro pipeline. 74% valid interactions were recognized in the WT group and 78% in the CKO group as shown in Fig. S7A. The normalized matrices were generated at 500 kb, 200 kb, 40 kb and 10 kb resolutions. The files generated after HiC-Pro processing were converted into “hic” files using Juicebox, and the contact maps were visualized using Juicebox [48]. A/B compartments were visualized using cworld-dekker scripts. TAD Compare was used to identify differential TAD boundaries and showed changes in TAD boundaries between two datasets [49].

### Statistical analysis

Quantification was performed in at least three independent experimental groups. The statistical analysis was carried out using SPSS v13. Two-tailed Student’s *t* test was used between the two groups to determine the significance, while one-way ANOVA with Tukey’s post-hoc test was used to compare differences between multiple groups. A *p* value of less than 0.05 was considered as significant, and error bars were presented as mean ± standard deviation (SD).

## Results

### Hnrnpk is expressed in developing limb buds and is essential for proper limb formation

To explore the role of Hnrnpk in developing limb bud, we first detected *Hnrnpk* expression in E10.5 to E12.5 embryos using whole-mount in situ hybridization (WISH) and quantitative polymerase chain reaction (qPCR) (Fig. 1A and Fig. S1A, B). At E10.5, *Hnrnpk* was expressed along the distal region of the limb bud, as well as in the midline and calvarium (Fig. 1A, top). Along with limb bud outgrowth, the *Hnrnpk* signal extended proximally to the central region of the limb bud at E11.5 (Fig. 1A, middle). Meanwhile, mesenchymal cells started to condense and express *Sox9*, and cartilage primordia of digits were found in the limb bud until E12.5 (Fig. 1A, *Sox9*-ref). As shown by WISH at E12.5, *Hnrnpk* was highly expressed in a primordia-like region and in the region of limb bud cells condensed, similar to *Sox9* expression (Fig. 1A, bottom). Taken together, the results demonstrated that *Hnrnpk* was expressed at early stage of developing limb buds, and then extended proximally, indicating a potential regulatory role of Hnrnpk in limb development.

To probe the regulatory function of Hnrnpk in embryonic limb buds, we engineered limb bud-specific *Hnrnpk* knockout mice [50], given the embryonic lethality of global *Hnrnpk* knockout (*Hnrnpk*^*−/−*^) mice [51]. We generated *floxed-Hnrnpk* mice (*Hnrnpk*^*fl/fl*^) using the embryonic cell targeting technique, which contained two *loxP* sites flanking exons 4–7 (Fig. 1B and Fig. S1C). This specific region was targeted because it is highly conserved across different transcripts and species (NCBI Reference Sequence: NM_001301341.1). We crossed *Hnrnpk*^*fl/fl*^ mice to *Prrx1-Cre* mice to generate *Prrx1-Cre;Hnrnpk*^*fl/fl*^ conditional knockout (CKO) mice, in which Hnrnpk expression was ablated in the limb bud and calvarium [52]. The efficient ablation of Hnrnpk was confirmed by western blotting (WB) analysis and qPCR of limb buds from E11.5 CKO embryos (Fig. 1C and Fig. S1D).

CKO mice died shortly after delivery, so we sacrificed the pregnant mice at E18.5 right before delivery. We observed a huge hematoma and encephalocele on the heads of CKO embryos (Fig. 1D, left panel and Fig. S2A), which might have caused lethality. Whole-mount skeletal preparation showed an absolute absence of forelimb and incomplete tiny hindlimb (Fig. 1D, right panels and Fig. S2B). The whole forelimb, from scapulae to phalanges, was missing, while the hindlimb was severely malformed although three components (stylopod, zeugopod, and autopod) were still recognizable in the CKO embryos (Fig. S2B). The differential phenotype of the forelimb and hindlimb might result from the incomplete expression of *Prrx1* in hindlimb [52]. Due to *Prrx1* expression in the calvarium, the calvarium of the CKO embryos also presented impaired craniofacial bone formation (Fig. S2A, bottom). To trace the key time point at which these defects occurred, we next examined the limb growth at E11.5-E13.5. A general inspection of CKO embryos at E11.5 showed no differences compared to wild type (WT) embryos (Fig. S2C). However, we hardly observed mesenchyme condensation in CKO embryos at E12.5 (Fig. 1E), which was the pivotal developmental window of digits. Until E13.5, CKO embryos presented absolutely absent forelimb skeletal elements (Fig. S2D).

Early in limb bud development, mesenchymal cells exhibit significant proliferation and partial apoptosis to form digits and joints properly [53]. To address whether cell death contributed to the limb deformities, we detected the apoptosis using a Lyso tracker. Excessive apoptosis was observed in the CKO embryos at E11.5 compared to less physiological apoptosis in WT embryos (Fig. 1F). Additionally, cultured primary limb bud cells and Ki67 immunostaining of E11.5 CKO embryos showed significant suppression of proliferation (Fig. S2E–G), and the TUNEL immunostaining of E11.5 CKO limb bud showed significant apoptosis (Fig. S2G). The results indicated that ablation of Hnrnpk in the limb bud resulted in impaired proliferation and excessive apoptosis of the limb bud mesenchymal cells.

Taken together, we demonstrated that Hnrnpk ablation in the limb bud led to the damaged survival and functioning of limb bud cells, and eventually resulted in the occurrence of limbless forelimbs and severe deformities in hindlimbs.

### Hnrnpk is required for the concordant regulation of master factors during embryonic limb bud development

**Fig. 2:**
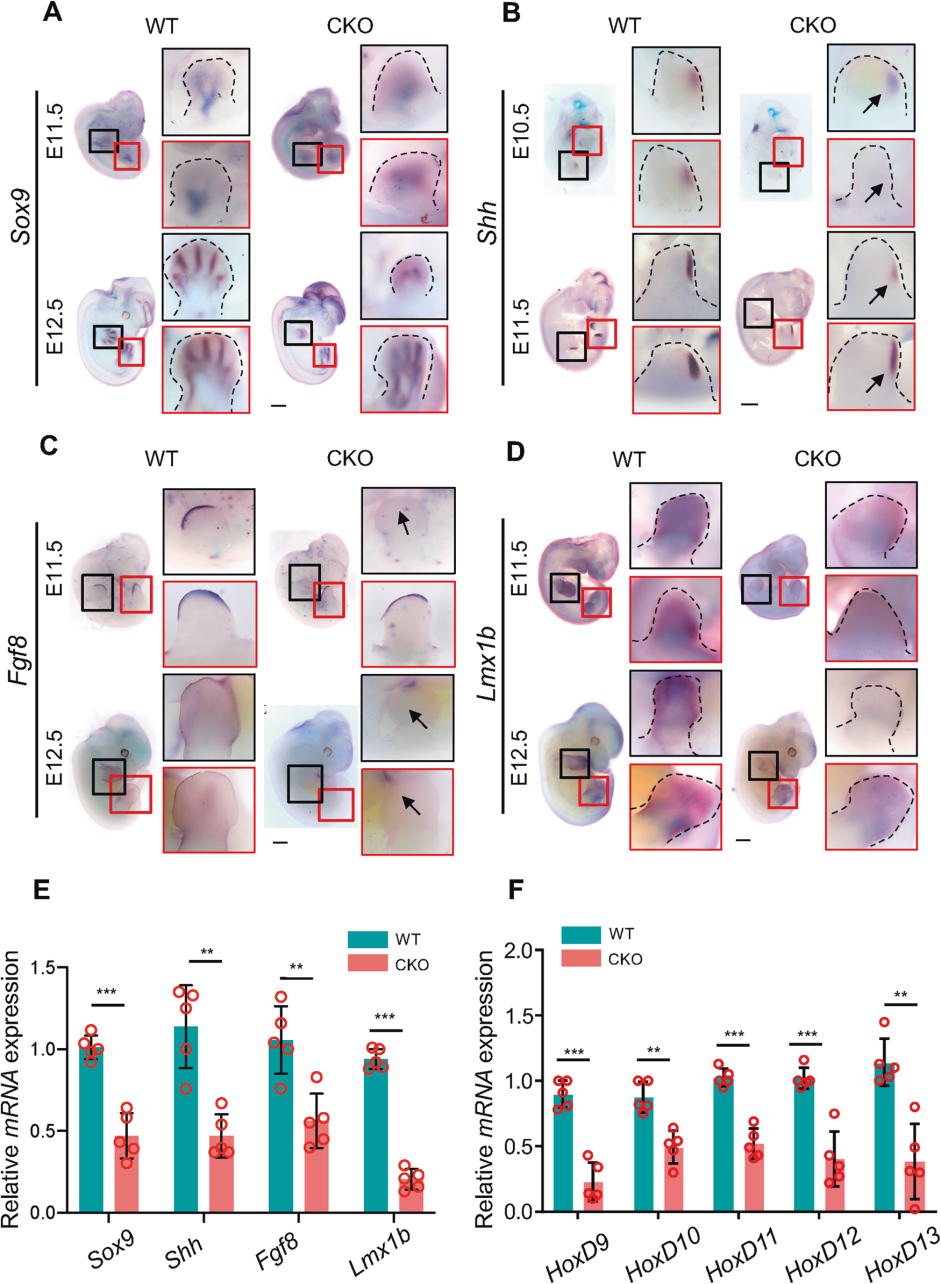
The regulatory axes of embryonic limb bud development are disturbed by Hnrnpk ablation. **A** Representative WISH images showed that *Sox9* failed to pattern along with the digits at E11.5 (top) and E12.5 (bottom) in CKO embryos compared to WT. The black boxes indicated the forelimbs, and the red boxes indicated the hindlimbs. Dotted lines indicated the limb buds. The magnification of limb buds was rotated to the same direction. Scale bar: 1 mm. **B** Representative WISH images showed the expression pattern and decreased signal intensity of *Shh* at E10.5 (top) and E11.5 (bottom) in CKO embryos compared to WT. The black boxes indicated the forelimbs, and the red boxes indicated the hindlimbs. Dotted lines indicated the limb buds. The arrows indicated the postaxial region of the limb buds. The magnification of limb buds was rotated to the same direction. Scale bar: 1 mm. **C** Representative WISH images showed vanished signals of *Fgf8* at the AER region at E11.5 (top) and E12.5 (bottom) in CKO embryos compared to WT. The black boxes indicated the forelimbs, and the red boxes indicated the hindlimbs. The arrows indicated the AER region. The magnification of limb buds was rotated to the same direction. Scale bar: 1 mm. **D** Representative WISH images showed decreased signal intensity of *Lmx1b* at the dorsal region of limb buds at E11.5 (top) and E12.5 (bottom) in CKO embryos compared to WT. The black boxes indicated the forelimbs, and the red boxes indicated the hindlimbs. Dotted lines indicated the limb buds. The magnification of limb buds was rotated to the same direction. Scale bar: 1 mm. **E** qPCR results confirmed the decreased expression of *Sox9*, *Shh*, *Fgf8*, and *Lmx1b* at E11.5 in CKO limb buds compared to WT. *N* = 5 biological replicates. **F** qPCR results showed that *HoxD9-13* were downregulated at E11.5 in CKO limb bud compared to WT. *N* = 5 biological replicates. The *p* value was calculated by two-tailed unpaired Student’s *t* test. Data were shown as mean ± SD. **p < 0.01, ***p < 0.001.

Normal limb bud growth relies on the concordant regulation of master factors, including SOX9, SHH, FGF8/10, LMX1B, and HOXD. Sox9 is expressed by differentiating chondrocytes at E11.5 and is considered as a marker of chondrogenesis. Based on WISH analysis, we found that CKO embryos exhibited dispersed, misaligned, and decreased *Sox9* expression at E11.5 and E12.5 in the forelimb (Fig. 2A). Similarly, the expression pattern of *Col2a1*, which is the predominant matrix in primordia, was misaligned at E11.5 and almost undetectable at E12.5 in CKO forelimb (Fig. S3A). To reveal whether Hnrnpk deficiency impaired the differentiation potential of limb bud mesenchymal cells, primary limb bud cells from E11.5 *Hnrnpk*^*fl/fl*^ embryos were isolated, subjected to micromass culture, and infected with adenovirus containing GFP (*Ad-GFP*) or Cre (*Ad-Cre*). The infected limb bud cells were then induced differentiated using a differentiation medium, followed by Alcian Blue staining at day 6 and Alizarin Red staining at day 12 (Fig. S3B). Hnrnpk was effectively knocked out after infection with *Ad-Cre* (Fig. S3C). The size of Alcian Blue-positive area decreased significantly after Hnrnpk ablated by *Ad-Cre*, whereas the Alizarin Red staining showed no significant differences between *Ad-GFP* and *Ad-Cre* (Fig. S3D). These results suggested that Hnrnpk deficiency impaired the chondrogenic potential of limb bud cells.

SHH is secreted by the ZPA region and regulates the proper formation of digits, which is the AP axis of limb development. Hnrnpk knockout resulted in weaker *Shh* expression at E10.5 and E11.5 in CKO limb buds, and no ectopic *Shh* expression was detected (Fig. 2B). *FGF8* is specifically expressed in the AER region and formed a positive regulatory loop with mesenchyme expressed *FGF10* to stimulate the elongation of limb bud, which is the PD axis of limb development [2]. Thus, we examined *Fgf8* expression by WISH and found that it was strictly expressed in the AER region, whereas the signals in the forelimb at E11.5 and both limb bud at E12.5 were hardly detected in the CKO embryos (Fig. 2C). Due to the absence of *Prrx1-Cre* recombinase activities in the AER region [54], where *Fgf8* is specifically expressed, Hnrnpk knockout driven by *Prrx1-Cre* would not directly affect *Fgf8* expression. However, except maintained via the Shh signal, Fgf8 is mainly triggered by Fgf10 expressed in limb bud mesenchyme, and we also detected decreased expression of *Fgf10* in CKO limb buds (Fig. S3E). As for the DV axis of limb development, Lmx1b is a master transcription factor expressed in the dorsal mesenchyme for the development of dorsal limb structures [55]. In the CKO embryos at E11.5 and E12.5, *Lmx1b* expression was much weaker in the dorsal limb bud, especially in the forelimb, compared to the WT embryos (Fig. 2D and Fig. S3F). The downregulation of key factors for the three axes and chondrogenesis were also confirmed by qPCR analysis using E11.5 CKO limb bud compared to WT (Fig. 2E). Lastly, the HOXD family is known to be sequentially activated in a rostrocaudal pattern, to guarantee accurate limb growth and polarity formation [13]. The expression of the *HoxD* family at E11.5 in CKO limb buds, including *HoxD9-13*, was significantly reduced (Fig. 2F), as indicated by qPCR analysis. Thus, our results demonstrated that Hnrnpk was required for the concordant and precise expression of vital molecules during embryonic limb bud development.

### Co-deletion of *Trp53* in CKO limb bud cells rescues apoptosis but not limb bud impaired development

**Fig. 3:**
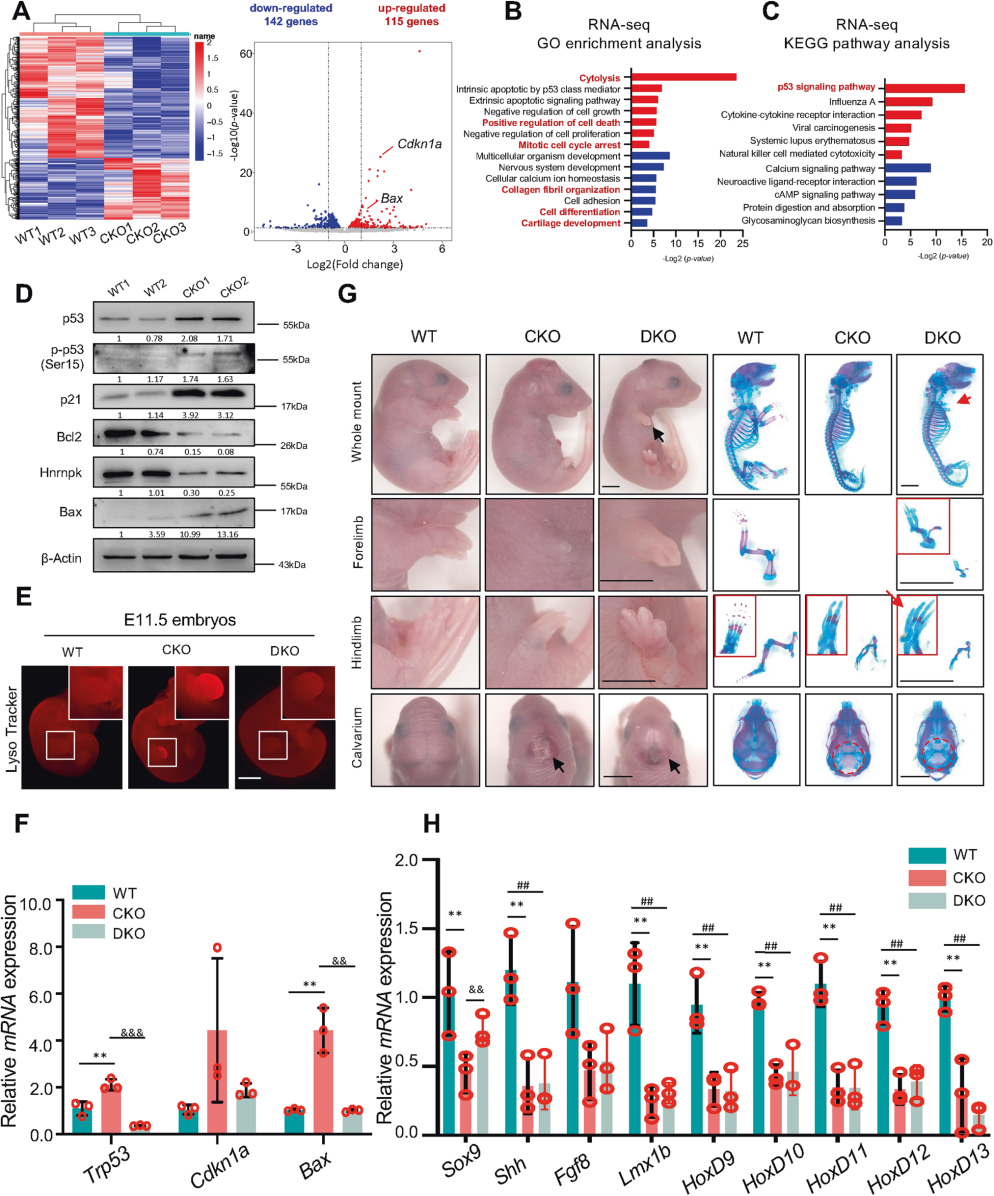
Knocking out *Trp53* in CKO mice is incapable of rescuing the phenotype. **A** Heatmap and volcano plot of RNA-seq using E11.5 WT and CKO limb buds. Three biological replicates were included. GO enrichment analysis (**B**) and KEGG pathway analysis (**C**) of differentially expressed genes (*p* value < 0.05) with fold change >2 of < 0.5. The items highlighted were the items we focus on. **D** Protein level of genes involved in the p53 signaling pathway, including p53, phosphate p53, p21, Bcl2, Hnrnpk, and Bax at E11.5 in WT and CKO limb buds. β-Actin as the loading control. Densitometry results were expressed as fold change in protein levels compared with WT1 after normalized to β-Actin. **E** Representative images of Lyso tracker staining of E11.5 WT, CKO, and DKO embryos. The white boxes indicated the magnification of the forelimbs. Scale bar: 1 mm. **F** qPCR results indicated the elevated RNA expression of *Trp53*, *Cdkn1a*, and *Bax* at E11.5 in CKO limb bud were reversed at E11.5 in DKO limb bud. *N* = 3 biological replicates. **G** Representative general observation and skeletal preparation of E18.5 WT, CKO, and DKO mice. The red boxes represented the magnification of the figure to show the details. The black arrows indicated the abnormal forelimbs and calvarium in DKO embryos. The red arrows indicated the partial recovery detected in DKO embryos. The red dotted circles indicated the missing calvarium. Scale bar: 1 mm. **H** qPCR results confirmed that decreased expression of *Shh*, *Fgf8*, *Lmx1b*, and *HoxD9-13* at E11.5 in CKO limb buds still existed at E11.5 in DKO limb bud. *N* = 3 biological replicates. The *p* value was calculated by one-way ANOVA with Tukey’s post-hoc test. Data were shown as mean ± SD. Statistical analysis was performed in WT and CKO embryos (indicated as **p < 0.01), or CKO and DKO embryos (indicated as ^&&^ *p* < 0.01, ^&&&^ *p* < 0.001), or WT and DKO embryos (indicated as ^##^ *p* < 0.01).

In order to figure out the molecular mechanism underlying Hnrnpk regulating limb bud development, we collected the limb buds from WT and CKO embryos at E11.5 for RNA-sequencing (RNA-seq, three biological replicates) to examine genome-wide gene expression changes in CKO limb buds. After mapping and annotation, 257 genes with a fold change >2 or <0.5 and a *p* value < 0.05 were filtered as differentially expressed genes compared to WT, consisting of 115 upregulated genes and 142 downregulated genes in CKO (Fig. 3A). The differentially expressed genes were subjected to Gene Ontology Biological Processes (GO-BP) analysis, which revealed that genes involved in organism development and cell differentiation were significantly downregulated, whereas genes associated with cell death were significantly upregulated (Fig. 3B), consistent with the findings described above.

The Kyoto Encyclopedia of Genes and Genomes (KEGG) pathway mapping showed that the activity of p53 signaling pathway was significantly upregulated (Fig. 3C). Increased p53 signal activity reportedly resulted in restriction of proliferation and excessive apoptosis, and eventually in skeletal deformity [56, 57]. To explore whether the phenotype observed in this study was caused by increased p53 signal activity, we initially confirmed the increased protein levels of p53, phosphorylated p53 (p-p53), and its downstream target genes, including Cdkn1a (also known as p21), and Bax. The anti-apoptotic factor Bcl2 was downregulated at E11.5 in CKO limb buds (Fig. 3D). We then knocked out *Trp53* (encoding p53 in mice) in CKO mice by mating them with *floxed-Trp53* mice to generate double knock-out mice (*Prrx1-Cre;Hnrnpk*^*fl/fl*^*;Trp53*^*fl/fl*^, DKO) (Fig. S4A). Knocking out *Trp53* in limb bud cells did not cause any developmental abnormality as previously reported [58], and the *Prrx1-Cre;Hnrnpk*^*fl/fl*^*;Trp53*^*fl/+*^ embryos showed no difference compared to the CKO embryos (Fig. S4B). However, the apoptotic signal in CKO embryos diminished significantly in DKO (Fig. 3E), and the activity of the p53 signaling pathway was reversed significantly in DKO embryos limb buds compared to CKO embryos (Fig. 3F), which indicated that the excessive apoptosis due to excessive p53 signaling pathway was significantly reversed. Although a small residue of forelimb skeletal elements was observed at E18.5, the DKO embryos were still exhibited severe deformity similar with CKO embryos (Fig. 3G). Except the expression of *Sox9* was partially rescued, the expression of other genes involved in limb bud development were still decreased at E11.5 in DKO limb buds compared to WT embryos and was undistinguishable from that of CKO embryos (Fig. 3H). Therefore, co-deletion of *Trp53* was incapable of rescuing the phenotype in CKO embryos, which also suggested that the excessive apoptosis caused by increased p53 signaling pathway was not the primary reason for the phenotype.

### Hnrnpk maintains gene expression in limb bud mainly through the transcriptional mechanism

**Fig. 4:**
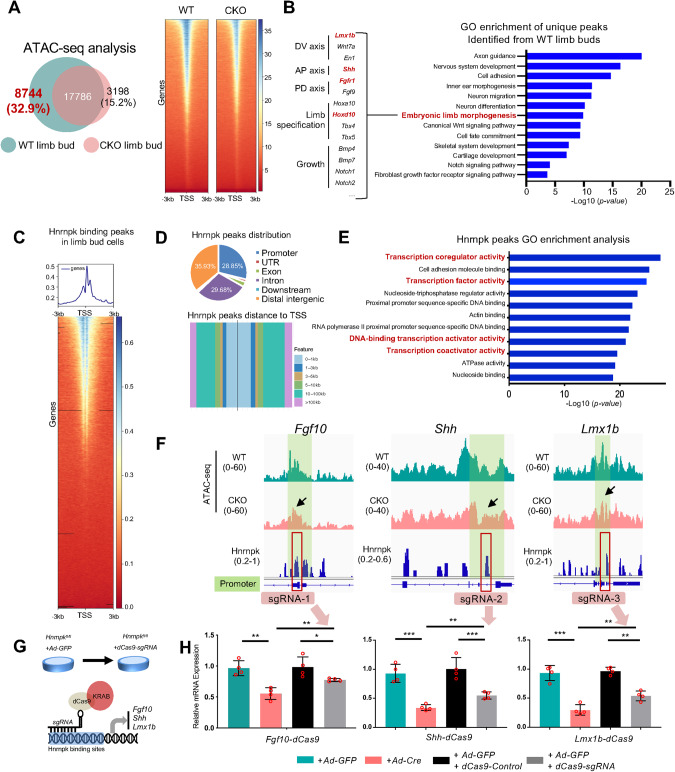
Hnrnpk maintains the regulation of regulatory axes in a transcriptional manner. **A** The number of peaks detected at E11.5 in WT and CKO limb buds by ATAC-seq, and signal intensities of peaks near transcriptional start sites (TSS). *N* = 3 biological replicates, replicates were merged. **B** GO enrichment analysis of genes upon the unique ATAC-seq peaks near TSS identified from WT limb buds compared to CKO. The items highlighted were the items we focus on. The genes involved in embryonic limb morphogenesis were shown on left. **C** Profile and heatmap of Hnrnpk CUT&RUN-seq performed with E11.5 WT limb buds. **D** The distribution and distances to the TSS of Hnrnpk binding peaks in the WT limb buds. **E** GO enrichment analysis of the Hnrnpk binding peaks in the WT limb bud. The items highlighted were the items we focus on. **F** ATAC-seq peaks and Hnrnpk binding at the TSS of *Fgf10-* (left panel)*, Shh-* (middle panel), and *Lmx1b*- (right panel) loci in WT and CKO limb buds. The green lines indicated the promoter regions. The red boxes indicated the target of the designed sgRNA. **G** Schematic diagram of constructing CRISPR/dCas9-KRAB vector. **H** qPCR results of the *Fgf10* (left panel), *Shh* (middle panel), and *Lmx1b* (right panel) in groups transfected *Hnrnpk*^*fl/fl*^ limb bud cells with *dCas9-control* or *dCas9-sgRNA* compared to *Hnrnpk*^*fl/fl*^ limb bud cells infected with *Ad-GFP* or *Ad-Cre*. *N* = 4 biological replicates. The *p* value was calculated by one-way ANOVA with Tukey’s post-hoc test. Data were shown as mean ± SD. *p < 0.05, **p < 0.01, ***p < 0.001.

To further explore the function of Hnrnpk at the transcriptional level, a genome-wide approach to determine the chromatin accessibility, assay for transposase-accessible chromatin using sequencing (ATAC-seq) was performed in WT and CKO limb bud cells (three biological replicates, data were merged). ATAC-seq showed that both the number of total peaks and peaks centered around the transcriptional start sites (TSS) decreased in the CKO limb buds (Fig. 4A and Fig. S5A, B). To illustrate transcriptional repression in absence of Hnrnpk, 8,744 unique peaks identified from WT embryos were subjected to GO enrichment analysis. Genes involved in the terms including embryonic limb morphogenesis, skeletal system development, and cartilage development were significantly enriched (Fig. 4B). Similar to the phenotype, the accessibility of promoters of genes involved in multiple aspects of limb bud development was repressed in the CKO limb bud (Fig. 4B) with decreased gene expression (Fig. S5C). Interestingly, the expression and chromatin accessibility of another paralogous group gene, *HoxA*, which is also vital during limb bud development [59], showed no significant change in the CKO limb bud (Fig. S5D, E).

Subsequently, to map genome-wide chromatin binding of Hnrnpk in limb buds, the cleavage under targets & release using nuclease with sequencing (CUT&RUN-seq) [45] was employed using Hnrnpk antibody in WT limb bud cells at E11.5 (Fig. 4C). Overall, 28.85% of the Hnrnpk-binding peaks located at promoters (Fig. 4D) and GO term mapping indicated that the Hnrnpk-binding peaks were highly related to transcription regulation, including transcription coregulator (Fig. 4E), suggesting a transcriptional regulatory role of Hnrnpk in mice limb buds. The motif analysis indicated that Hnrnpk exhibited binding preference for multi-cytosine-patch (Fig. S5G), which similar to its binding preference in RNA [60]. Combining ATAC-seq with CUT&RUN-seq results, we found that the accessibility of *Fgf10*, *Shh*, and *Lmx1b* promoter regions decreased significantly in CKO limb buds, and prominent Hnrnpk-binding peaks were visible in these regions (Fig. 4F). To test whether Hnrnpk regulated the transcription of *Fgf10*, *Shh*, and *Lmx1b*, the binding of Hnrnpk in their promoter regions were functionally inhibited by placing a nuclease-deficient(d) Cas9-Krüppel associated box (KRAB) fusion protein (dCas9-KRAB) over a narrow range (~300 bp) of corresponding Hnrnpk-binding sites using guide RNAs via a lentivirus (Fig. 4G and Fig. S5H). Targeting dCas9-KRAB to promoters recruits the H3K9 methyltransferase SET domain bifurcated histone lysine methyltransferase 1 (SETDB1), which increases local H3K9me3 binding and represses target gene expression [61]. Primary limb bud cells were cultured and infected with *Ad-GFP, Ad-Cre*, lentivirus-dCas9 (*dCas9-Control*), or lentivirus-dCas9-sgRNA (*dCas9-sgRNA*), followed by qPCR analysis. As expected, the expression of *Fgf10*, *Shh*, and *Lmx1b* in limb bud cells was significantly reduced by Hnrnpk knockout (*Ad-Cre*) or Hnrnpk-binding site blockade (*dCas9-sgRNA*) (Fig. 4H), confirming that Hnrnpk transcriptionally maintained the proper expression of these master factors during limb bud development. Nevertheless, the expression decreases induced by *dCas9-sgRNA* were always significantly lower than those caused by *Ad-Cre* (Fig. 4H, pink and gray columns), suggesting that other mechanisms underlying Hnrnpk regulating the expression of *Fgf10*, *Shh*, and *Lmx1b* existed.

### Hnrnpk ablation weakens interactions between promoters and enhancers

**Fig. 5:**
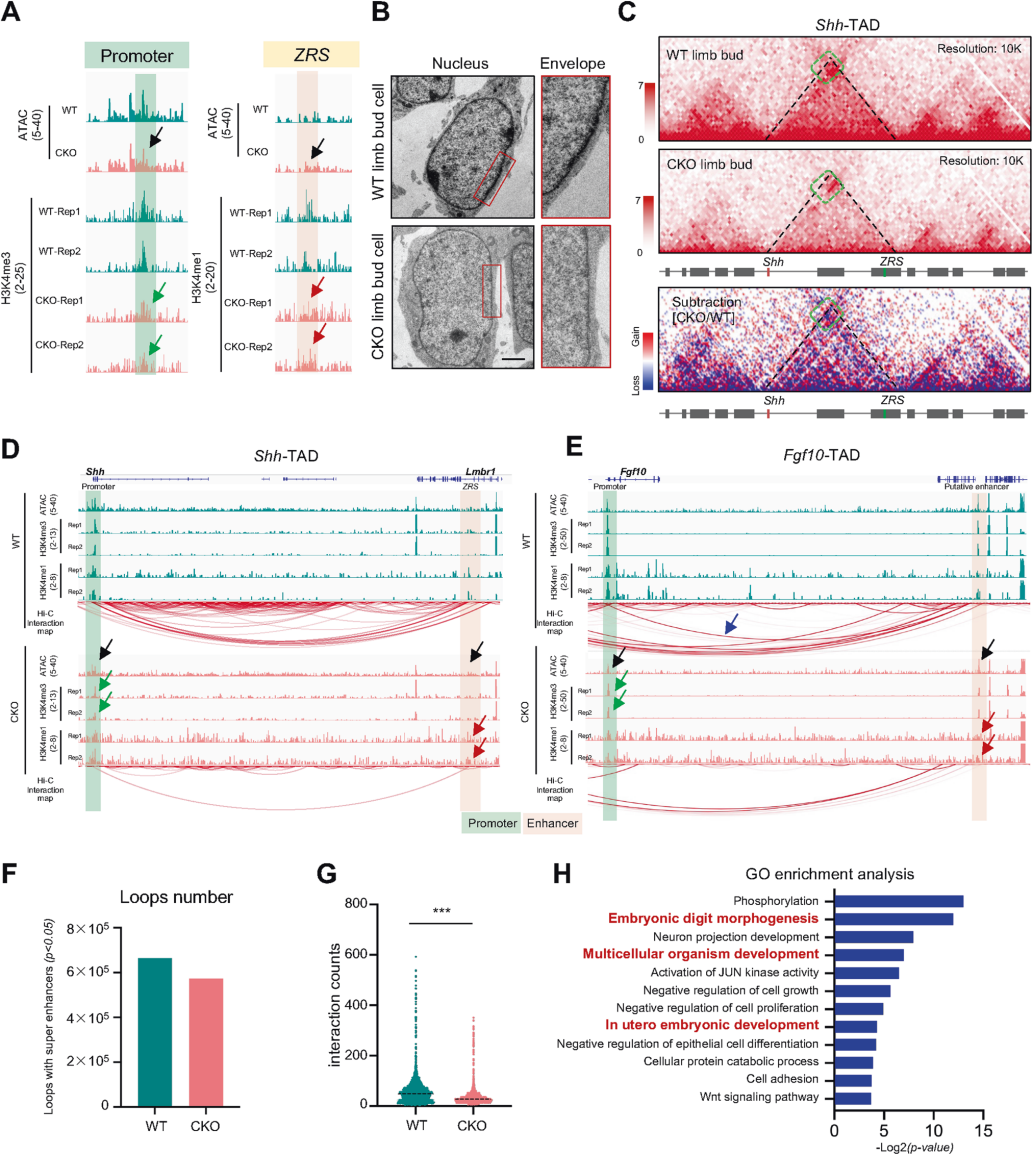
Hnrnpk ablation weakens the interactions between promoters and enhancers. **A** Genome browser tracks of ATAC-seq data, H3K4me3 and H3K4me1 CUT&RUN-seq data at *Shh* promoter and *Shh* enhancer *ZRS* at E11.5 in WT and CKO limb buds. The black arrows indicated decreased ATAC peaks. The green arrows indicated decreased H3K4me3 peaks in the promoter regions of the CKO groups. The red arrows indicated decreased H3K4me1 peaks in the enhancer regions of the CKO groups. **B** Representative electron microscope images of E11.5 WT and CKO limb bud. The red boxes indicated the magnification of the nuclear envelope. Scale bar: 2 μm. **C** Hi-C heatmap at *Shh-*TAD. The green boxes indicated the contact heatmap between *Shh* and *ZRS* at E11.5 in WT and CKO limb bud cells. **D**, **E** ATAC-seq data, H3K4me3, and H3K4me1 CUT&RUN-seq data, and Hi-C interaction map at *Shh-* (**D**) and *Fgf10-* (**E**) TADs. The black arrows indicated decreased ATAC peaks in the CKO groups. The green arrows indicated decreased H3K4me3 peaks in the promoter regions of the CKO groups. The red arrows indicated decreased H3K4me1 peaks in the enhancer regions of the CKO groups. The blue arrow indicated the interaction between *Fgf10* and its putative enhancer. **F** Quantity of loops detected at E11.5 in WT and CKO limb bud. **G** Average loop interaction intensity at E11.5 in WT and CKO limb buds. **H** GO enrichment analysis of target genes of loop interaction intensity decreased more than 2 folds (Log_2_FC < −1) detected at E11.5 in CKO limb buds compared to WT. The *p* value was calculated by two-tailed unpaired Student’s *t* test. Data were shown as mean ± SD. ***p < 0.001.

Previous studies have indicated that a three-dimensional chromatin structure helps the spatial approach of the distal enhancer to the proximal promoter within one TAD [24, 62, 63]. Thus, we proposed that Hnrnpk might regulate the expression of *Shh*, *Fgf10*, *Lmx1b*, and *HoxD* partially via a three-dimensional genome approach, in addition to direct interactions with promoter regions. To this end, we mapped the binding of H3K4me3 (indicating activated promoters) and H3K4me1 (indicating enhancers and promoters) at E11.5 in WT and CKO limb buds using CUT&RUN-seq. Consistent with the results described earlier, the binding intensity of H3K4me3 in TSS on the whole genome decreased significantly (Fig. S6A), which proved the repressive transcription activities in CKO limb bud cells. As with the transcriptional repression in the CKO limb buds, the intensities of the H3K4me3-binding peaks significantly decreased in the *Shh* promoter region in the CKO limb buds (Fig. 5A). Interestingly, the binding intensities of H3K4me1 in intron 5 of *Lmbr1* (containing *ZRS*) were impaired, and the chromatin accessibility of this region was reduced, as indicated by ATAC-seq in the CKO limb buds (Fig. 5A). These results indicated the impaired function of enhancers in the absence of Hnrnpk during embryonic limb growth.

To study the mechanism, we tested the expression of H3K4me1, H3K4me3, and H3K27ac (indicating enhancers and promoters) but no significant change detected in CKO limb bud compared to WT (Fig. S6B), and except H3K4me3, the direct interaction between Hnrnpk and H3K4me1 or H3K27ac was also weak (Fig. S6C). We also compared the overlap of binding between Hnrnpk and H3K4me1, H3K4me3, and H3K27ac (GSE151487) [64], and the results showed that the overlap between Hnrnpk and H3K4me1/H3K27ac was less significant than H3K4me3 (Fig. S6D), suggesting that Hnrnpk did not affect the function of enhancers directly.

Interactions between cis-regulatory elements, especially long-distance interactions, depend on the proper chromatin three-dimensional structure. Therefore, we explored the chromatin structure using electron microscope imaging to determine the reason for the impaired function between cis-regulatory elements. The nuclear envelope of the CKO limb bud cells was more diffused and looser than that of the WT limb bud cells (Fig. 5B), suggesting that some changes of chromatin architecture occurred. To confirm the changes in chromatin architecture, we isolated the E11.5 limb bud cells and mapped the interaction using Hi-C assay (Fig. S7A). Hi-C data provided the information on the three-dimensional chromatin architecture. We compared the interactions between WT and CKO among *Shh-*TAD, and the decreased interactions between *Shh* and *ZRS* were detected in CKO limb bud cells (Fig. 5C, green boxes). From the viewpoint of *Shh* locus, we further assessed the intensity of loops, the physical association between distal enhancer and target promoter [65]. We found decreased intensity of the loop connecting *Shh* promoter and *ZRS* in CKO limb bud cells, indicating the interactions between *Shh* promoter and *ZRS* were damaged (Fig. 5D). Similar changes were observed in the *Fgf10*-locus (Fig. 5E) and *HoxD*-locus (Fig. S7B). Totally, in the whole genome, there was 18.2% of TADs changed significantly in CKO (Fig. S7C). We also found that the number of loops with super enhancers of CKO limb bud cells decreased significantly comparing with loops of WT limb bud cells (Fig. 5F), and average interaction intensity also decreased (Fig. 5G). We then selected the loops whose interaction intensity decreased more than twofold and subjected them to GO enrichment analysis. We found that the terms including embryonic digit morphogenesis, organism development, and embryonic development were significantly enriched (Fig. 5H), which indicated that some genes involved in limb bud development were repressed due to the weakened interaction between promoters and enhancers. Thus, our data demonstrated that ablation of Hnrnpk resulted in significant changes in chromatin structure and, subsequently, interactions between regulatory elements.

### Hnrnpk ablation weakens the binding strength of Ctcf at TAD boundaries

**Fig. 6:**
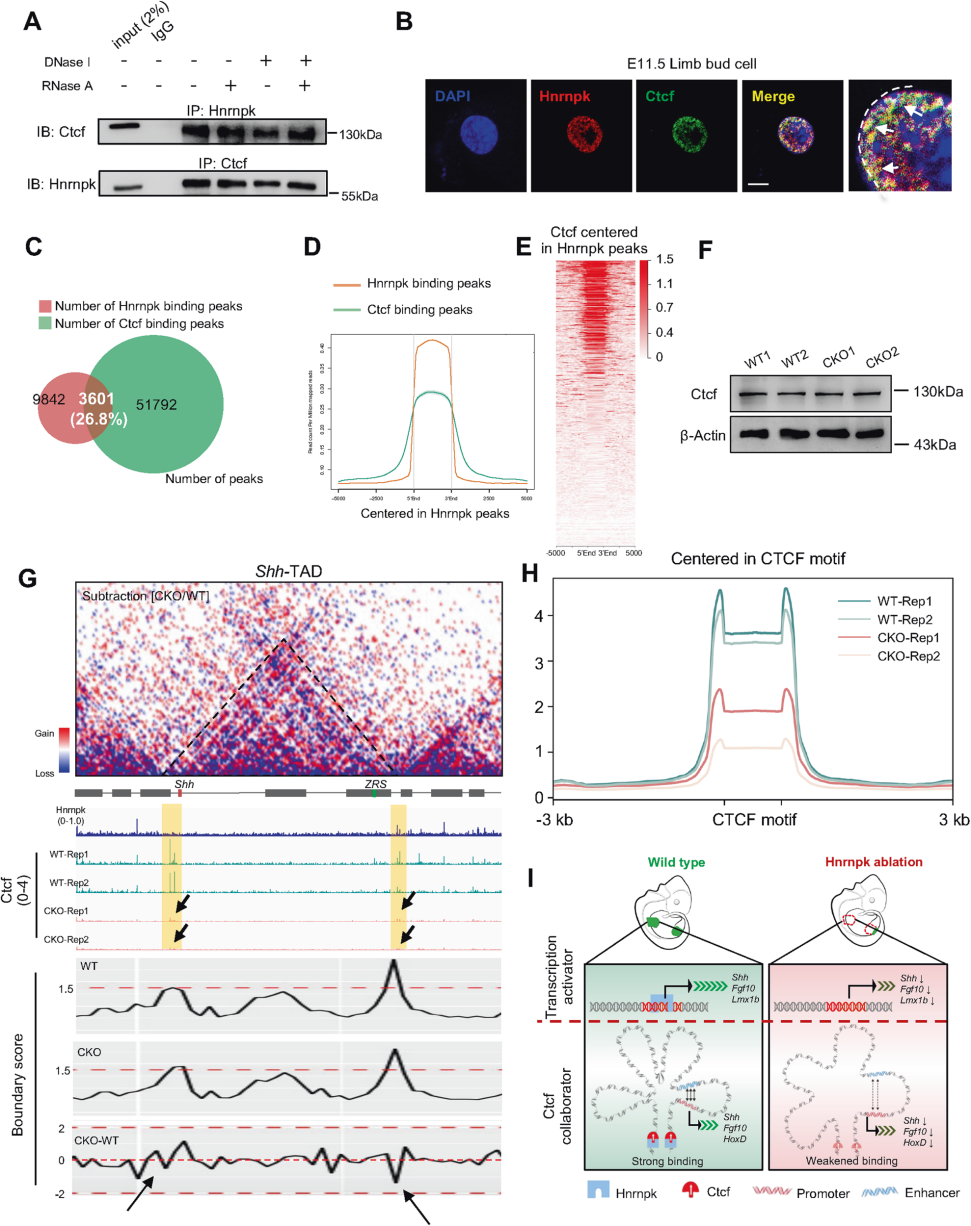
Hnrnpk weakens the binding strength of Ctcf at TAD boundaries. **A** Co-Immunoprecipitation (co-IP) assay between Hnrnpk and Ctcf at E11.5 in WT limb bud after treatment with PBS, DNase I, RNase A, or both. **B** Subcellular location of Hnrnpk and Ctcf at E11.5 in primary WT limb bud cell. The white arrows indicated the overlapped Hnrnpk and Ctcf signals in the nucleus. The white dotted line indicated the margin of nucleus. Scale bar: 10 μm. **C** The Hnrnpk- and Ctcf- binding peaks at E11.5 in WT limb buds and their overlap. The ratio indicated the proportion of overlapped peaks in the total peaks of Hnrnpk. **D**, **E** The binding profile of Ctcf centered in Hnrnpk binding peaks. **F** Protein level of Ctcf at E11.5 in WT and CKO limb buds. β-Actin was used as the loading control. **G** Hi-C contact map (upper) and genome browser tracks of Hnrnpk and Ctcf CUT&RUN-seq data (bottom) at *Shh*-TAD. The black arrows indicated the decreased binding strength of Ctcf at E11.5 in CKO limb bud. The yellow lines indicated the boundaries at both sides of *Shh-*TAD. **H** The Ctcf binding peaks centered in CTCF-motif at E11.5 in WT and CKO limb buds. **I** A proposed model illustrated the role of Hnrnpk as a transcription activator and insulator protein collaborator, which regulated the transcription of key regulatory genes during embryonic limb bud development.

To explore the reason for the change in chromatin structure, we performed motif analysis using WT unique peaks detected in ATAC-seq, and the results showed that CTCF and CTCF-like (Boris) were enriched most significantly (Fig. S8). CTCF is a kind of insulator protein regulating the chromatin architecture. Considering the chromatin structure changes in CKO limb bud cells and the large proportion (35.93%) of Hnrnpk binding sites located in distal intergenic regions (Fig. 4D), we proposed that Hnrnpk might play a role in coordinating with Ctcf to regulate chromatin remodeling during embryonic limb development. To test our hypothesis, we performed co-immunoprecipitation (co-IP) assays using primary limb bud cells and found that Hnrnpk bound to Ctcf, which was independent of DNA or RNA (Fig. 6A). The extensive expression of Hnrnpk in limb bud cell nucleus was further verified with a laser scan confocal microscope. Dual staining with Ctcf revealed that the signal of Hnrnpk significantly overlapped with Ctcf in the nucleus (Fig. 6B). Functionally, 26.8% of Hnrnpk-binding peaks overlapped with Ctcf-binding peaks in WT limb buds at E11.5 according to the CUT&RUN-seq results (Fig. 6C), and Hnrnpk peaks were significantly enriched for Ctcf (Fig. 6D, E).

To illustrate whether the changes in the three-dimensional chromatin architecture were associated with the functional changes of Ctcf in the CKO limb bud, we first examined the expression of Ctcf after ablating Hnrnpk, but no significant difference was detected (Fig. 6F). Then, we mapped the binding sites of Ctcf in WT and CKO limb bud cells to detect the binding strength of Ctcf and combined the outcome with the binding map of Hnrnpk. The results showed that Hnrnpk bound to the boundary regions of *Shh*-TAD, and the binding strength of Ctcf at the boundaries of *Shh*-TAD also decreased remarkably in the absence of Hnrnpk, resulting in a decrease in the boundary score of TAD (Fig. 6G). Similar changes in TAD and its boundaries were found in *Fgf10*-TAD (Fig. S9A). The *HoxD*-locus is located between two adjacent TADs: the centromeric domain (C-DOM) and the telomeric domain (T-DOM). In E11.5 limb bud cells, *HoxD12-13* mainly interacted with C-DOM, while *HoxD9-11* interacted with both C-DOM and T-DOM [13, 66, 67]. We also detected the decreased interactions in both TADs and decreased Ctcf binding strength at their boundaries in CKO limb bud cells (Fig. S9B). Unexpectedly, the *Lmx1b-*TAD of CKO limb bud cells did not display obvious changes, especially no changes in Ctcf binding strength, which might be explained by the slight interactions within this *Lmx1b-*TAD at E11.5 (Fig. S9C). Compared to the Ctcf binding map in WT limb buds, a significant decreased in Ctcf binding strength around CTCF-motif was detected in CKO limb buds (Fig. 6H and Fig. S9D, E). Taken together, our data demonstrated that Hnrnpk ablation in limb bud cells reduced the Ctcf binding strength to TAD boundaries and impaired the interactions inside TAD.

## Discussion

In this study, we demonstrated that specific ablation of Hnrnpk in the limb bud led to severe developmental defects, exhibiting limbless forelimbs and severe deformities of hindlimbs with oligodactyly. The phenotype involved the abnormal development of three axes. We previously reported that increased binding of hnRNPK to the mutant *ZRS* resulted in ectopic and overexpression of *SHH* and occurrence of PPD-I [34]. Nonetheless, the model did not illustrate how hnRNPK helps *ZRS* approach the *SHH* promoter spatially. Here, we found that Hnrnpk ablation led to reduced chromatin accessibility in both *Shh* promoter and enhancer, which further resulted in decreased *Shh* expression. Of note, *Shh* knockout embryos (*Shh*^*−/−*^) had four limbs with recognizable humerus/femur bones [68, 69], which was less severe than that of Hnrnpk CKO mice, suggesting that Hnrnpk ablation damaged limb bud development beyond the AP axis. In addition to the disruption of the AP axis, Hnrnpk ablation also disturbed the PD axis and DV axis. Only disruption of the PD axis by *Fgf10* knockout resulted in limb truncation with rudimentary scapulae and pelvis remaining [70], whereas no scapulae were observed in absence of Hnrnpk. The partial loss of hindlimb in CKO embryos might result from the incomplete ablation of Hnrnpk by *Prrx1-Cre* [52]. Thus, the ablation of Hnrnpk resulted in the overall disruption of all three regulatory axes, suggesting a fundamental regulatory role of Hnrnpk in the early-stage limb bud. Additionally, it has been reported that hnRNPK functions as a co-activator of p53 under DNA damage and knocking down hnRNPK decreases the activity of the p53 signaling pathway [71]. Different from the previous study, we detected increased activity of the p53 signaling pathway and the upregulated expression of its downstream target genes. The results indicated that the elevated activity of the p53 signal might be a secondary change due to the functional failure of limb bud cells, rather than a primary change after knocking out Hnrnpk.

The RNA-seq and ATAC-seq results exhibited widespread downregulation of developmental genes expression and chromatin accessibility. In this study, we mapped the Hnrnpk binding in limb bud cell and the peaks were detected in promoter regions, while more significant binding was detected in both up and down stream of TSS than TSS. We speculated that the bimodal signal of Hnrnpk around TSS was relevant to its function coupling with RNA polymerase II (RNA POL II), which was widely reported before in multiple cell types [29, 71, 72], and we also detected the same phenomenon in limb bud cell (Fig. S10A, B). RNA POL II possessed the character of promoter-proximal pausing, which eluded detection by conventional ChIP due to its experimental principle [73]. CUT&RUN was more precise with higher resolution so that the bimodal signal was detected in CUT&RUN data of RNA POL II [74]. Thus, the similar bimodal signal we detected in Hnrnpk CUT&RUN might be due to its functional coupling with RNA POL II. However, only 28.85% of the Hnrnpk-binding peaks were located in the promoter regions, and a remarkable proportion (35.93%) were located in the distal intergenic regions. These results suggested that Hnrnpk exceeded the role of a transcription factor in regulating early-stage limb bud development. A loose chromatin structure was detected by electron microscope in CKO limb bud cells, which indicated that Hnrnpk participated in regulating chromatin architecture. Using Hi-C assay, we detected global remodeling of chromatin architecture, with a significantly decreased intensity of loop between *Shh* promoter and *ZRS*. Similar changes were also detected between the *HoxD* promoters and the enhancers. Although hnRNPK was supposedly associated with chromatin remodeling during the progression of prostate cancer [75, 76], and for the first time, our results showed direct evidence that the three-dimensional chromatin architecture was disrupted in the absence of Hnrnpk in the limb bud.

Similar to the changes in *Shh-*locus and *HoxD*-locus, decreased interactions between the *Fgf10* promoter and its putative enhancer were also detected in the *Fgf10*-locus. In limb bud cells at E11.5, *Shh* possessed *ZRS* as its certain distal enhancer [63], and at this stage, *HoxD* possessed multiple distal enhancers, referred to as Conserved sequences B (CsB) and Prox [77, 78] in C-DOM and Conserved noncoding sequences 39 and 65 (CNS 39 and 65) in T-DOM [67] (Fig. S7B). However, the distal enhancer of *Fgf10* in mice limb bud has not yet been identified. A few enhancers of mice limb buds had been reported, but most of them were located around the promoter of *Fgf10* [79]. There was a distal enhancer located at 233 kb downstream from the promoter of *Fgf10* in chick limb buds [80], although our data did not show enhancer activity at this location, which might result from differences in species or developmental stages. Interestingly, we detected a putative enhancer located 700 kb downstream from the promoter and their strong interactions (Fig. 5E, blue arrow). Thus, our data suggested that there might be a distal enhancer of *Fgf10* in mice limb buds, which deserved further authentication.

Among the chromatin structures, TAD is crucial for orchestrating different functional units [16], which are regulated by pivotal insulator protein CTCF [81]. Previous studies have demonstrated that the disturbance of CTCF binding in *Shh-*TAD resulted in up to 50% decreased expression of *Shh* [24]. Similarly, deleting CTCF binding sites around the *HoxD* cluster also resulted in the decreased expression of some *HoxD* genes [25]. In our study, we found that Hnrnpk was located mainly in limb bud cell nuclei and bound to Ctcf, independent of DNA or RNA. Functionally, decreased Ctcf binding strength at boundaries flanking the TADs of *Shh*, *Fgf10*, and *HoxD* in CKO limb bud cells was indicated, confirming that Hnrnpk maintained a three-dimensional chromatin architecture by regulating Ctcf. Similarly, another component of the hnRNP family, hnRNPU, was previously identified as a regulator of TAD formation, but the results showed that ablation of hnRNPU impaired the binding strength of RAD21, instead of CTCF [82]. In summary, our study revealed the novel role of Hnrnpk as a collaborator of the insulator protein Ctcf.

Interestingly, during the embryonic limb bud development, the function as an insulator protein that CTCF acted was dispensable, even though a moderate decrease of genes expression was detected when the isolation was disturbed [83]. Thus, in our study, the phenotype of Hnrnpk null mice was caused by a superimposed mechanism of decreased transcription and decreased chromatin interaction.

However, the mechanism by which Hnrnpk regulated Ctcf binding strength was still unknown. Notably, RNAs have been proven to be involved in three-dimensional chromatin architecture regulation [84], and RNA binding region deletion mutant CTCF reduces its binding strength at boundaries and fail to shape TADs [85]. Long noncoding RNA *Colorectal Cancer Associated Transcript 1-long* (*CCAT1-L*) reportedly interacts with CTCF in the *MYC* locus and regulates chromatin conformation in colorectal cancer cells [86], and recently, a study demonstrated that hnRNPK interacted with *CCAT1-5L* RNA and regulated the formation of a super-enhancer complex, using RNA in situ conformation sequencing (RIC-seq) [72]. In our study, Hnrnpk bound to Ctcf independent of RNA, and the ablation of Hnrnpk did not affect the expression of Ctcf. However, the manner how Hnrnpk ablation impaired the Ctcf binding strength at the boundaries has not yet been determined. Non-coding RNAs might be involved in and regulate the complex of hnRNPK and CTCF at TAD boundaries, which deserved further study.

Figure 6I summarized our findings, showing that Hnrnpk was essential for limb bud development by regulating vital gene expression, and that its ablation resulted in limbless and other skeletal deformities. In terms of mechanism, we demonstrated that a dual regulatory role for Hnrnpk during embryonic limb development. For the first time, we revealed the novel role of Hnrnpk as an insulator protein collaborator in regulating global three-dimensional chromatin architecture, beyond a transcription activator. Ablation of Hnrnpk resulted in decreased binding strength of Ctcf at chromatin boundaries, leading to the loose TADs.

### Supplementary information

Supplementary materials

Supplementary materials Western blot

## Data Availability

The RNA-seq data, ATAC-seq data, CUT&RUN-seq data, and Hi-C data have been uploaded to GEO database with the number GSE186411.
