# Supplementary material for: Hnrnpk is essential for embryonic limb bud development as a transcription activator and a collaborator of insulator protein Ctcf
Source: Cell Death Differ. 2023 Aug 22;30(10):2293–308. doi: 10.1038/s41418-023-01207-z (PMC10589297; doi:10.1038/s41418-023-01207-z)
Supplement: Supplementary file 1 — Supplementary materials [file 41418_2023_1207_MOESM1_ESM.docx]

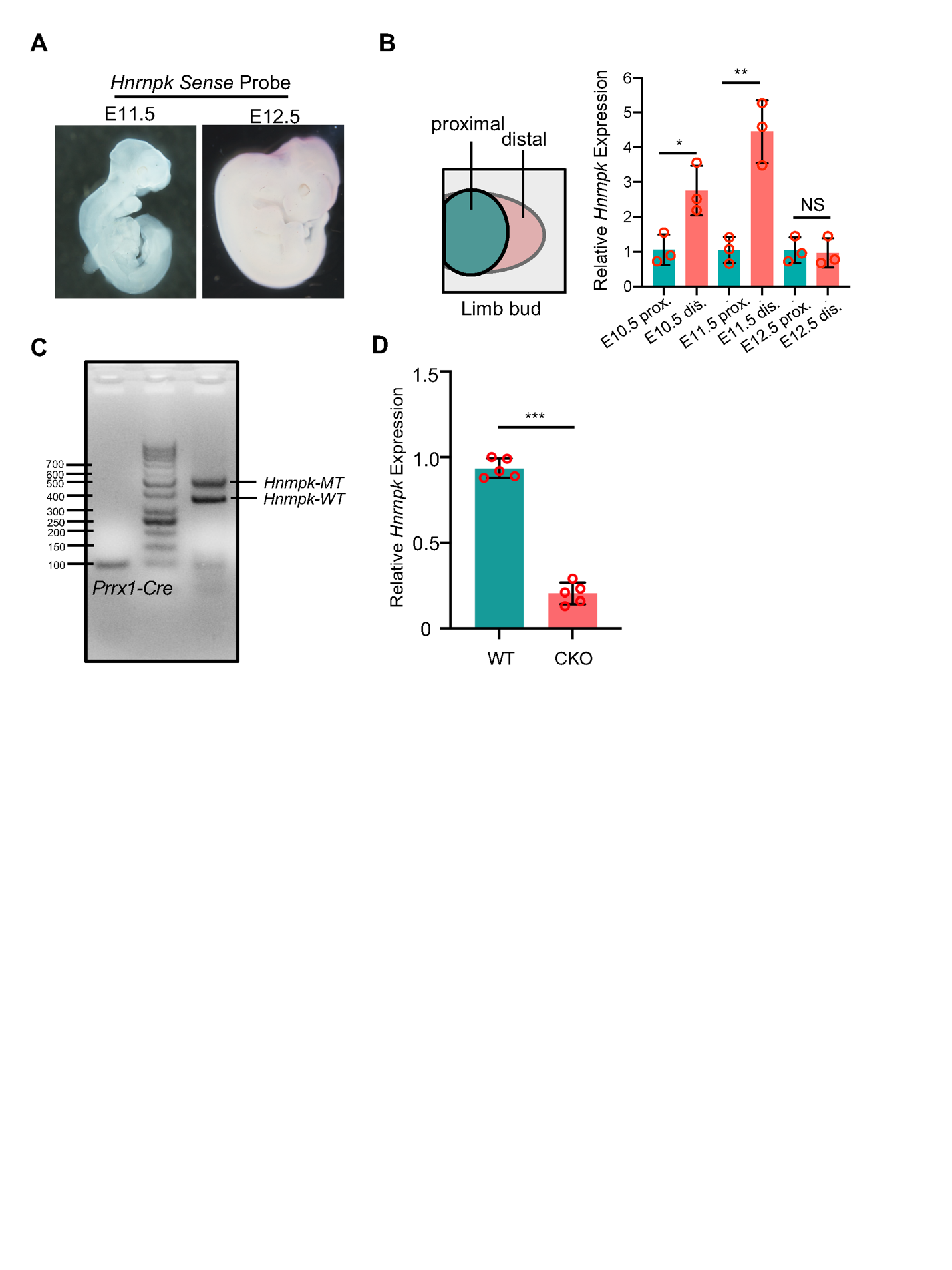


**Fig. S1. *Hnrnpk* is expressed extensively in the developing limb buds.**

A. WISH using *Hnrnpk­* sense probe as the negative control. Scale bar: 1 mm.

B. qPCR results of proximal and distal part of limb bud at E10.5, E11.5, and E12.5. Prox.,proximal. Dis., distal. *N* = 3 biological replicates.

C. Gel images showed the genotypes of *Prrx1-Cre* and *Hnrnpk-loxP*. *Hnrnpk-WT:* 360bp; *Hnrnpk-MT:* 473bp; *Prrx1-Cre:* 100bp.

D. qPCR results verified the efficient knockdown of *Hnrnpk* at E11.5 in CKO limb buds compared to WT. *N* = 5 biological replicates.

The *p*-value was calculated by two-tailed unpaired Student’s *t*-test. Data were shown as mean ± SD. **p* < 0.05, ***p* < 0.01, ****p* < 0.001. NS, not significant.

**
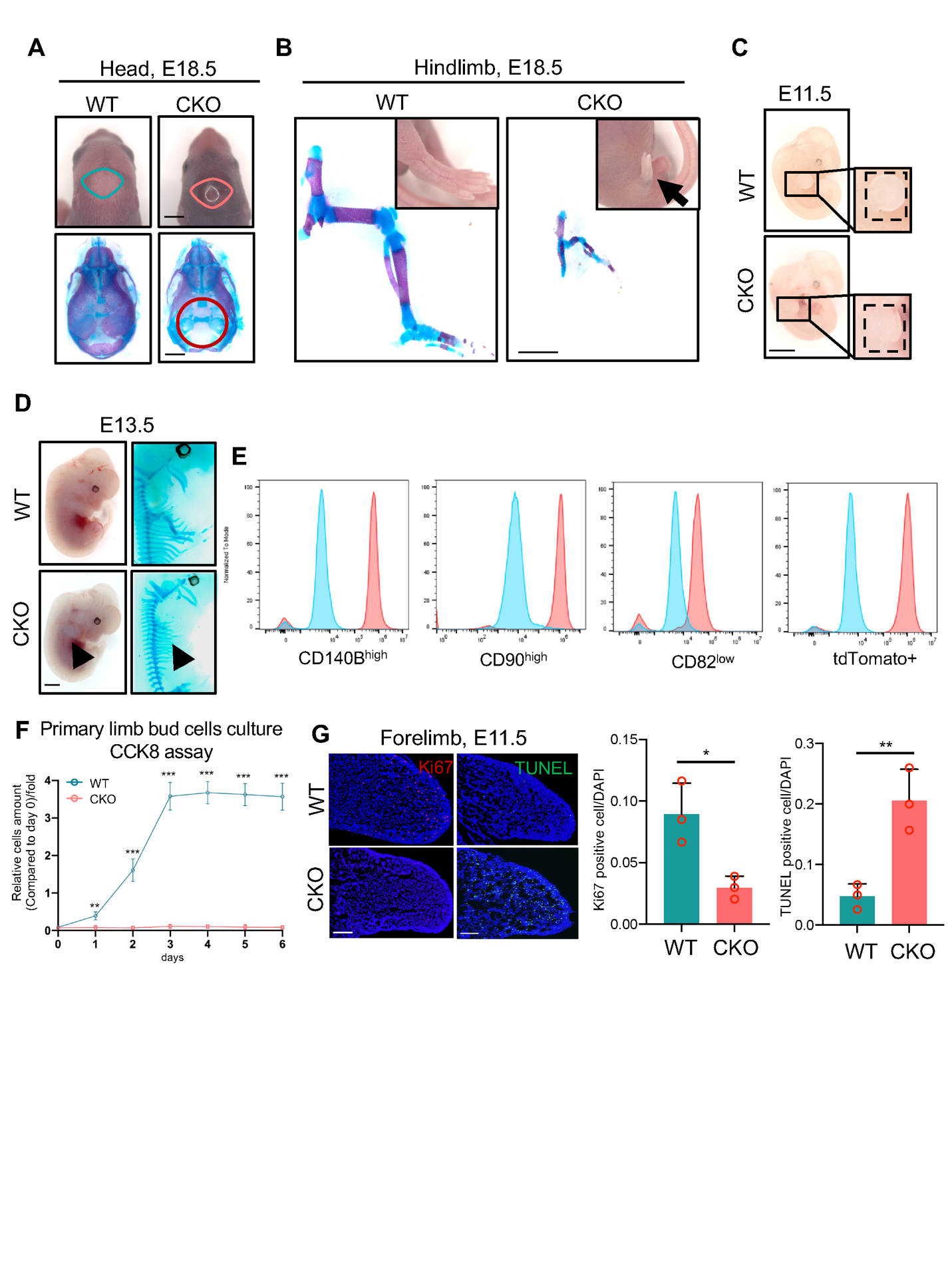
**

**Fig. S2. Loss of Hnrnpk results in limb deformities and skull defects.**

A. Representative images of the heads of E18.5 WT and CKO embryos. The calvarium outline was indicated by the green line for WT and the pink line for CKO (upper). Skeletal preparation showed the severe craniofacial bone formation defects in the CKO embryos. The missing calvarium was indicated by the red circle (bottom). Scale bar: 1 mm.

B. Representative general observation and skeletal preparation images of E18.5 WT and CKO hindlimbs. Arrow indicated the oligodactyly in hindlimb of the CKO embryos. Scale bar: 1 mm.

C. Representative general observation images of E11.5 WT and CKO embryos. Scale bar: 1 mm.

D. Representative general observation (left panel) and skeletal preparation (right panel) images of E13.5 WT and CKO embryos. The arrows indicated the residual soft tissue and the total absence of skeletal element in CKO embryo forelimb. Scale bar: 1 mm.

E. The flow cytometric analysis of primary limb bud cells.

F. The proliferative curve of the primary limb bud cells was depicted using CCK8 assay. *N* = 4 biological replicates.

G. Representative immunofluorescent staining images using Ki67 antibody and TUNEL assay at E11.5 in WT and CKO forelimbs and their quantifications. *N* = 3 biological replicates. Scale bar: 100μm.

The *p*-value was calculated by two-tailed unpaired Student’s *t*-test. Data were shown as mean ± SD. **p* < 0.05, ***p* < 0.01, ****p* < 0.001.


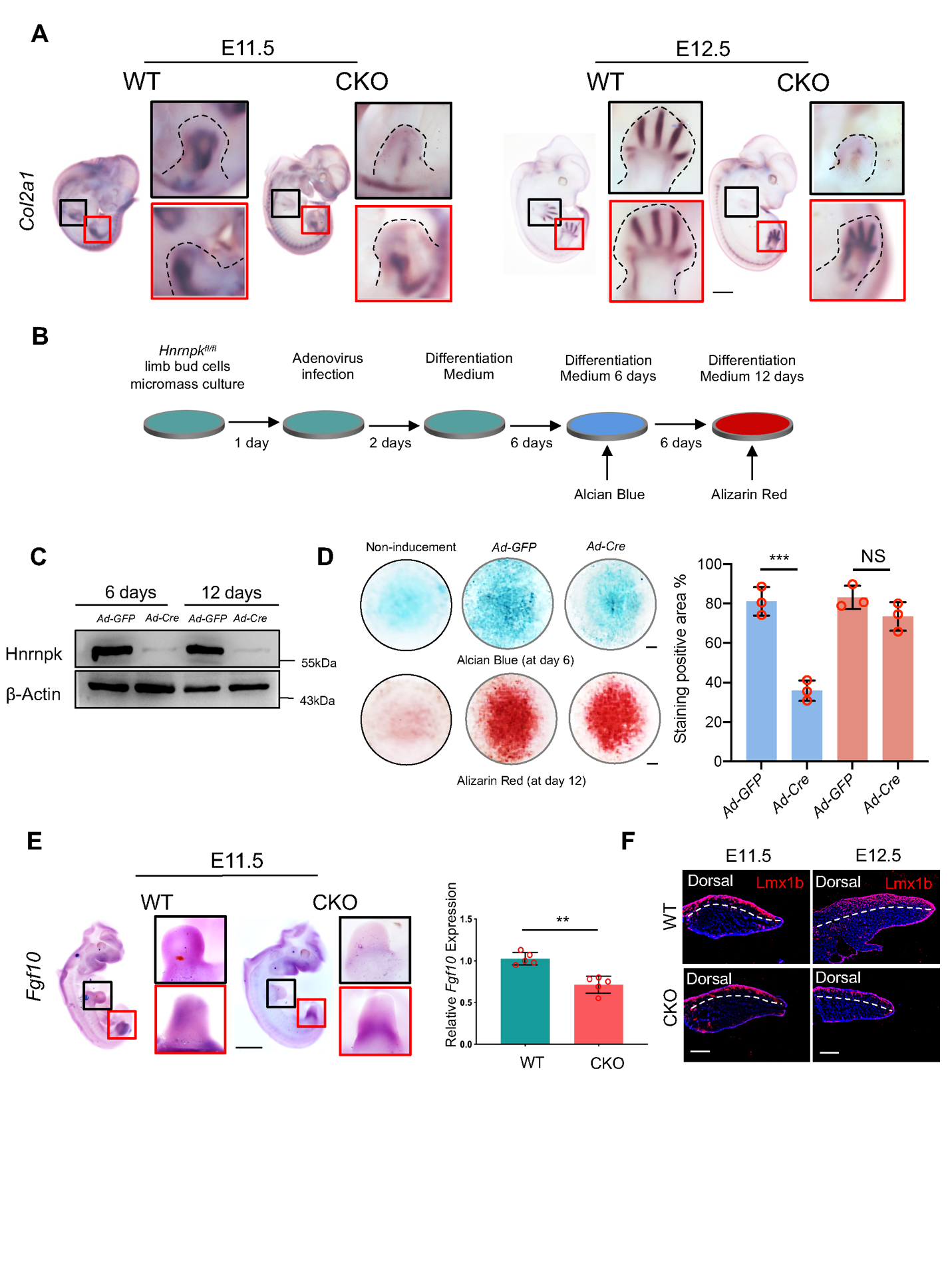


**Fig. S3. Loss of Hnrnpk damages the chondrogenic potential of the limb bud cells.**

A. Representative WISH images showed the weaker *Col2a1* signals at E11.5 in CKO limb bud compared to WT and the shrunken signals at E12.5 in CKO limb bud. The black boxes indicated the forelimbs, and the red boxes indicated the hindlimbs. Dotted line indicated the limb bud. Scale bar: 1 mm.

B. Diagram of the Alcian Blue staining and Alizarin Red staining after micromass culture of *Hnrnpk^fl/fl^* limb bud cells infected with *Ad-GFP* or *Ad-Cre*.

C. WB indicated the efficient knock down at day 6 and day 12 in *Hnrnpk^fl/fl^* limb bud cells infected with *Ad-Cre*.

D. Representative images of the Alcian Blue staining (upper) at day 6 and the Alizarin Red staining (bottom) at day 12 and their quantifications. *N* = 3 biological replicates. Scale bar: 500 μm.

E. Representative WISH images (left panel) and qPCR results (right panel) showed that *Fgf10* was significantly decreased at E11.5 in CKO limb buds. The black boxes indicated the forelimbs, and the red boxes indicated the hindlimbs. *N* = 5 biological replicates. Scale bar: 1 mm.

F. Representative immunofluorescent staining images using Lmx1b antibody at E11.5 and E12.5 in WT and CKO forelimbs. The upper part of images was the dorsal limb bud and the lower part was ventral limb bud. Scale bar: 100 μm.

The *p*-value was calculated by two-tailed unpaired Student’s *t*-test. Data were shown as mean ± SD. ***p* < 0.01, ****p* < 0.001. NS, not significant.

**
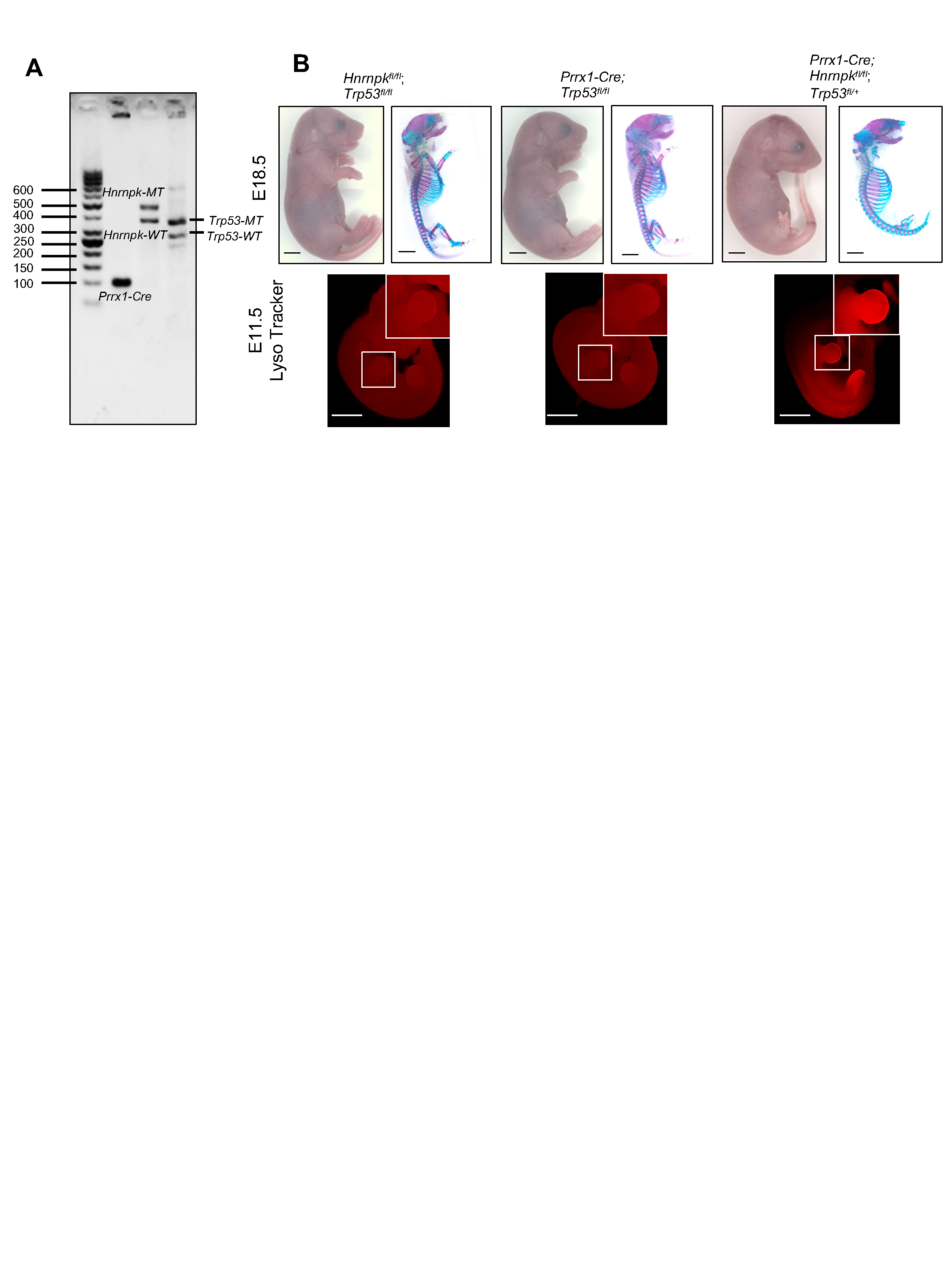
**

**Fig. S4. Activation of the p53 signaling pathway is secondary to failed limb bud development.**

A. Gel images showed the genotypes of *Prrx1-Cre*, *Hnrnpk-loxP*, and *Trp53-loxP*. *Hnrnpk-WT*: 360bp; *Hnrnpk-MT*: 473bp; *Prrx1-Cre*: 100bp; *Trp53-WT*: 270bp; *Trp53-MT*: 360bp.

B. Representative general observation and skeletal preparation of E18.5 (upper) and the Lyso tracker staining of E11.5 (bottom) in *Hnrnpk^fl/fl^;Trp53^fl/fl^*, *Prrx1-Cre;Trp53^fl/fl^* , and *Prrx1-Cre;Hnrnpk^fl/fl^;Trp53^fl/+^* embryos. Scale bar: 1 mm.

**
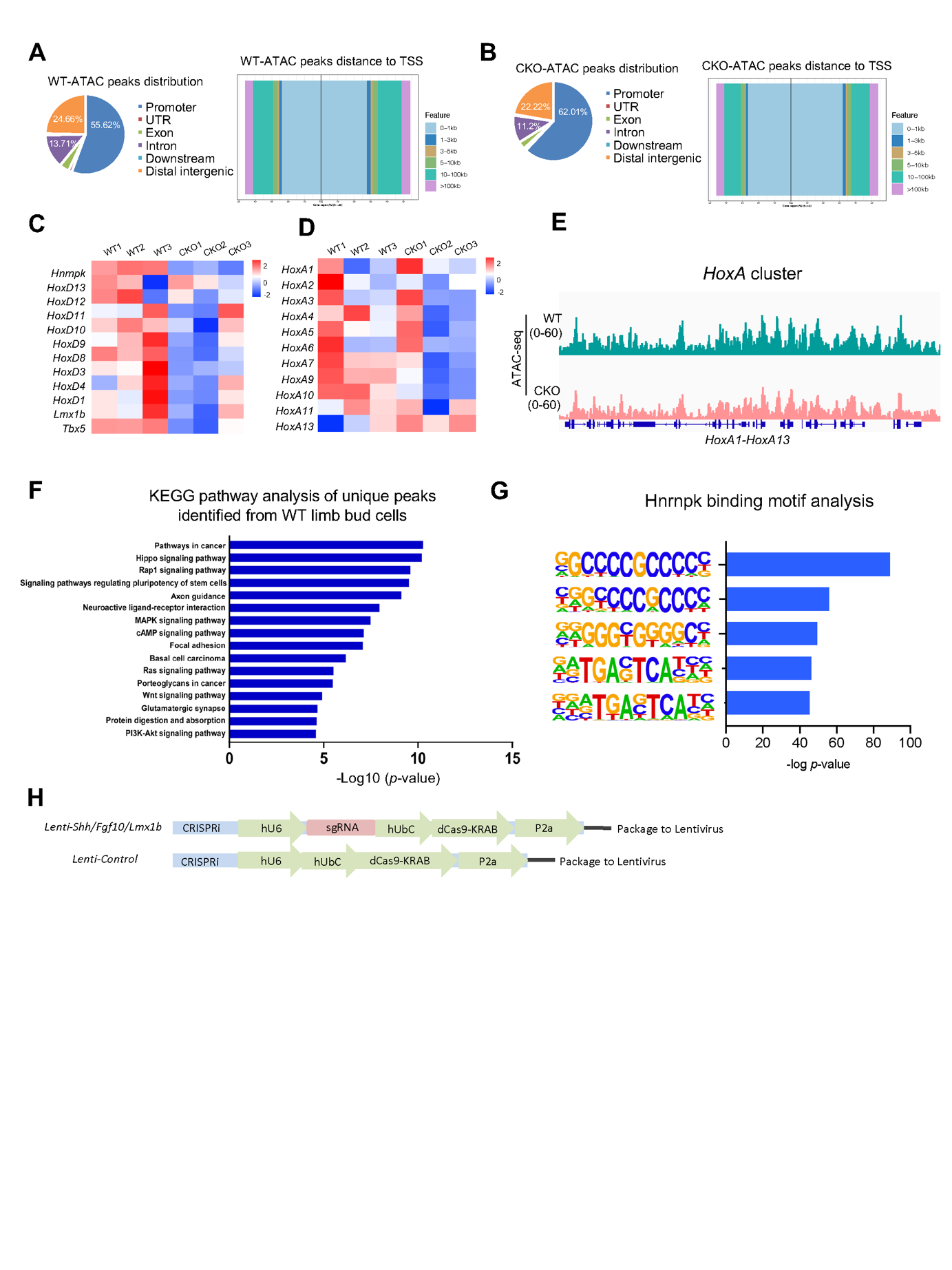
**

**Fig. S5 Hnrnpk regulates genes expression as transcription activator.**

A-B. The distribution of ATAC peaks of WT (A) and CKO (B) limb bud cells.

C. Decreased expression of genes involving limb bud developments according to the RNA-seq results. *N* = 3 biological replicates.

D. The gene expression of the *HoxA* cluster showed no significant difference according to RNA-seq results. *N* = 3 biological replicates.

E. Genome browser tracks of ATAC-seq data at the *HoxA*-locus in WT and CKO limb bud cells.

F. KEGG pathway analysis of the unique ATAC-seq peaks near TSS identified from WT limb buds compared to CKO.

G. Motif analysis of Hnrnpk binding peaks identified from WT limb bud cells.

H. Linearized maps of the CRISPR/dCas9-KRAB plasmid.


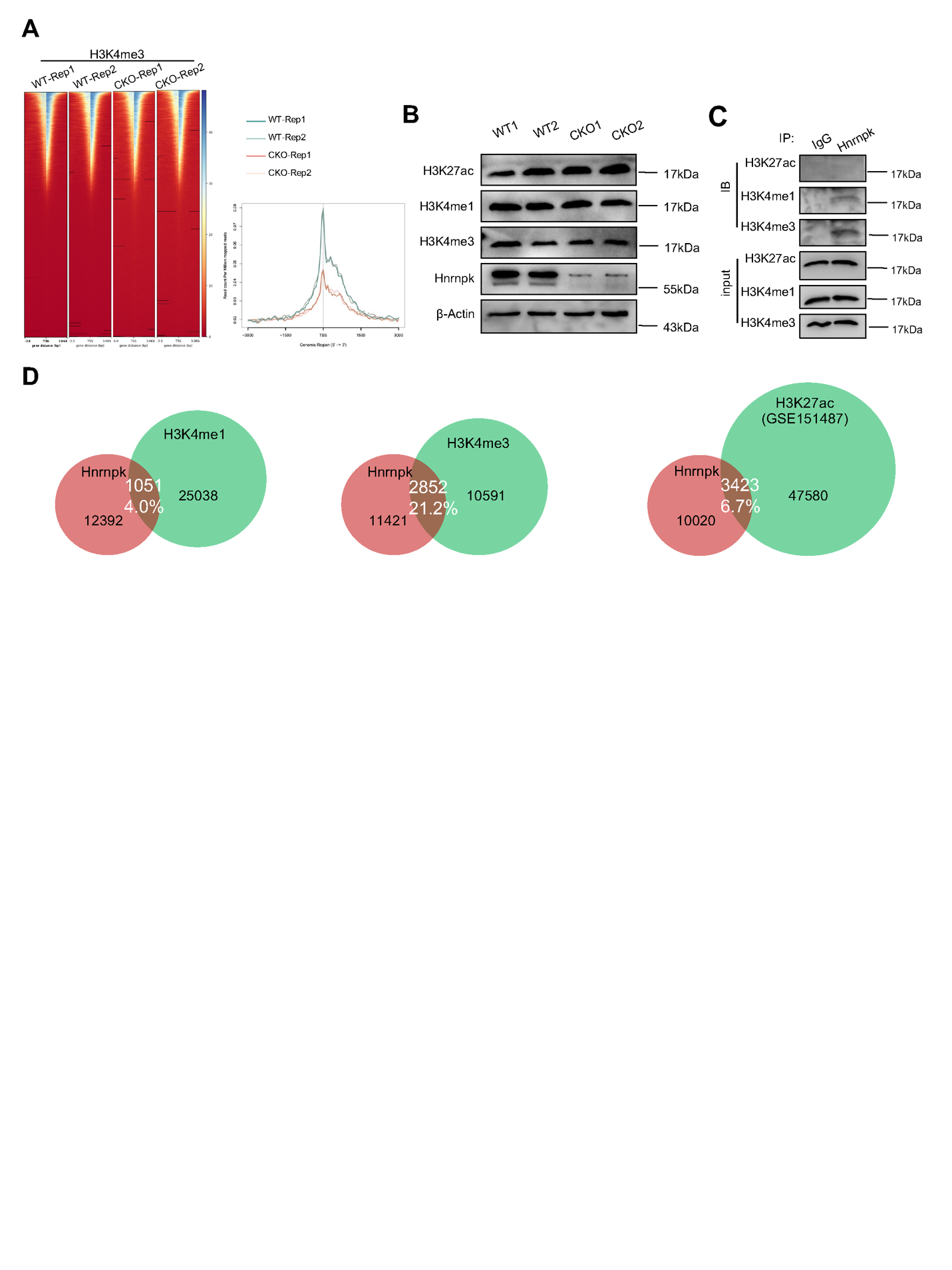


**Fig. S6. Hnrnpk ablation results in damaged function of enhancer.**

A. The heatmap and profile of H3K4me3 binding peaks at E11.5 in WT and CKO limb buds.

B. The expression of H3K27ac, H3K4me1, and H3K4me3 at E11.5 in WT and CKO limb buds.

C. IP assay between Hnrnpk and H3K27ac, H3K4me1, or H3K4me3 at E11.5 in WT limb buds.

D. The quantity and ratio of overlapping peaks between Hnrnpk binding peaks and H3K4me1, H3K4me3, or H3K27ac at E11.5 WT limb bud cells.

**
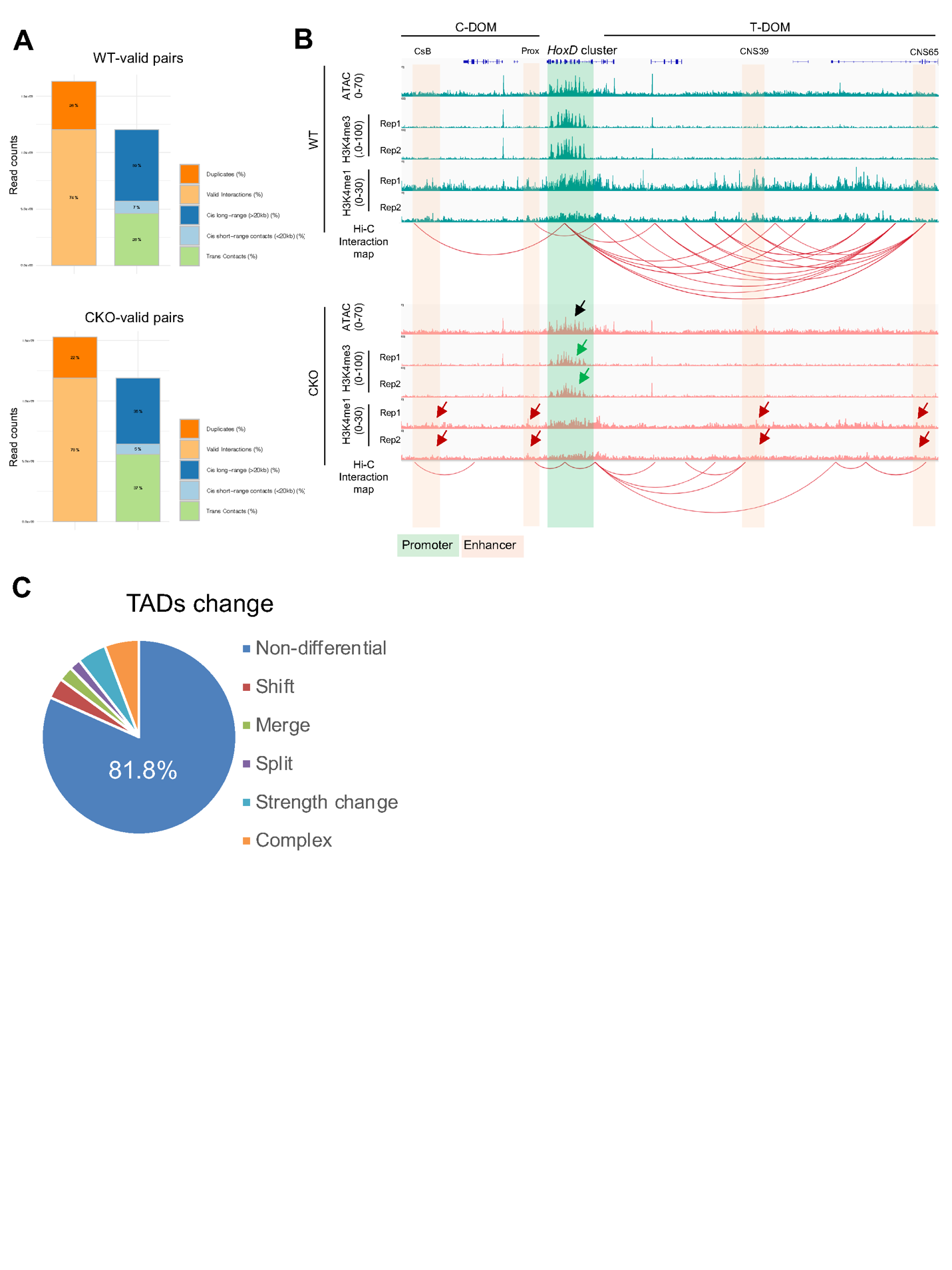
**

**Fig. S7. Hnrnpk ablation results in the change of chromatin architecture.**

A. Valid pairs identified at E11.5 in WT and CKO limb bud cells.

B. Genome browser tracks of ATAC-seq data, H3K4me3, H3K4me1 CUT&RUN-seq data, and Hi-C interaction map at the *HoxD*-locus in WT and CKO limb bud cells. The black arrows indicated the decreased ATAC peaks in promoter regions of the CKO groups. The green arrows indicated the decreased H3K4me3 peaks in promoter regions of the CKO groups. The red arrows indicated the decreased H3K4me1 peaks in the enhancer regions of CKO groups. C-DOM: Centromeric domain. T-DOM: Telomeric domain.

C. The change of types, quantity, and ratio of TADs in CKO limb bud cells compared to WT limb bud cells.

**
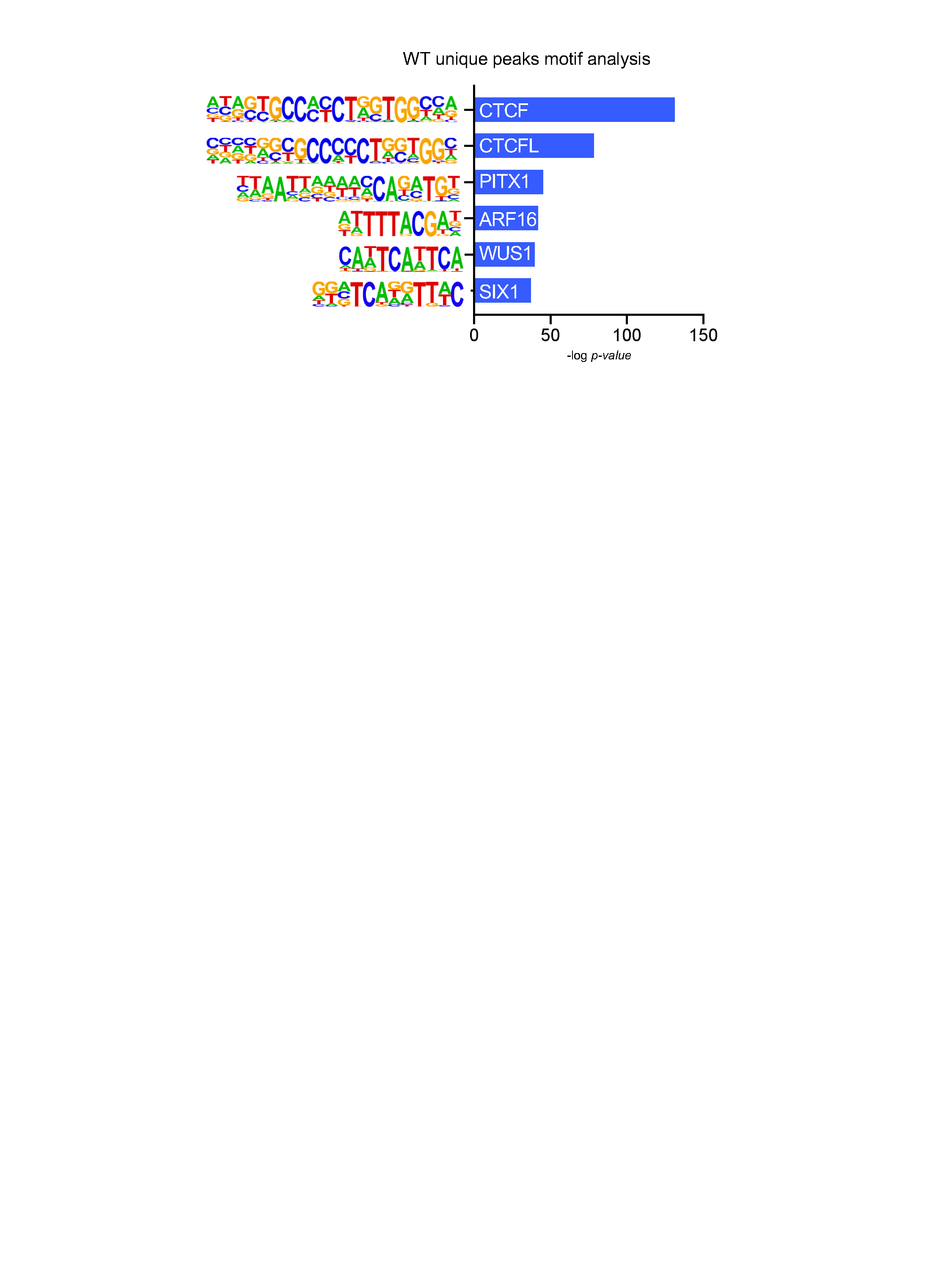
Fig. S8. Hnrnpk regulates chromatin architecture formation via cooperating with Ctcf.** Motif analysis using WT unique peaks detected in ATAC-seq.

**
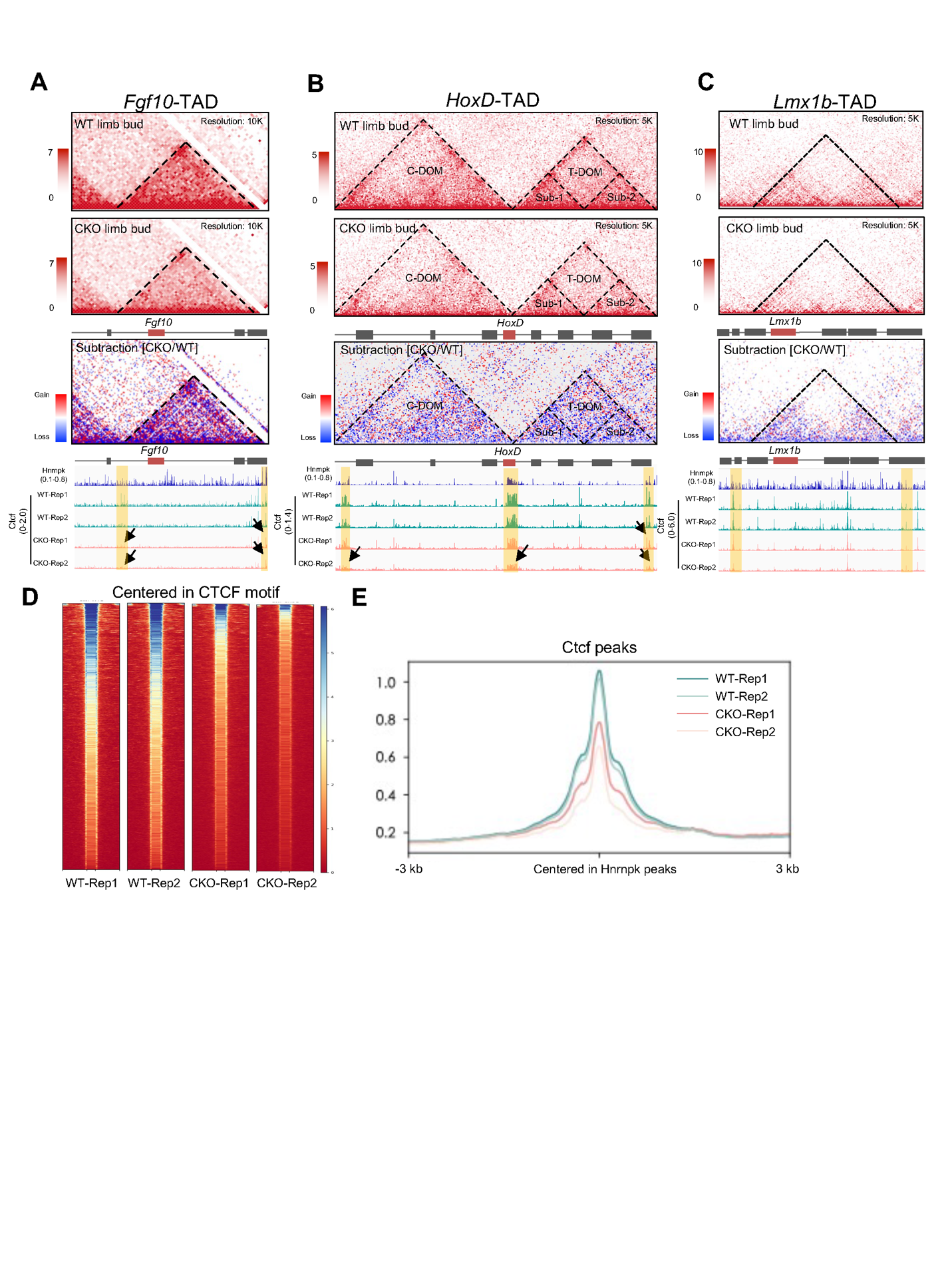
**

**Fig. S9. Hnrnpk cooperates with Ctcf to regulate TAD formation.**

A-C. Hi-C contact map (upper) and genome browser tracks of Hnrnpk and Ctcf CUT&RUN-seq data (bottom) at *Fgf10-* (A), *HoxD-* (B), and *Lmx1b-* (C) TADs. The black arrows indicated the decreased binding strength of Ctcf in boundaries. Yellow line indicated boundaries of TADs. C-DOM: Centromeric domain. T-DOM: Telomeric domain.

D. CUT&RUN-seq signal intensities of Ctcf around CTCF motif at E11.5 in WT and CKO limb buds.

E. The binding profile of Ctcf of WT and CKO limb bud centered in Hnrnpk binding peaks.

**
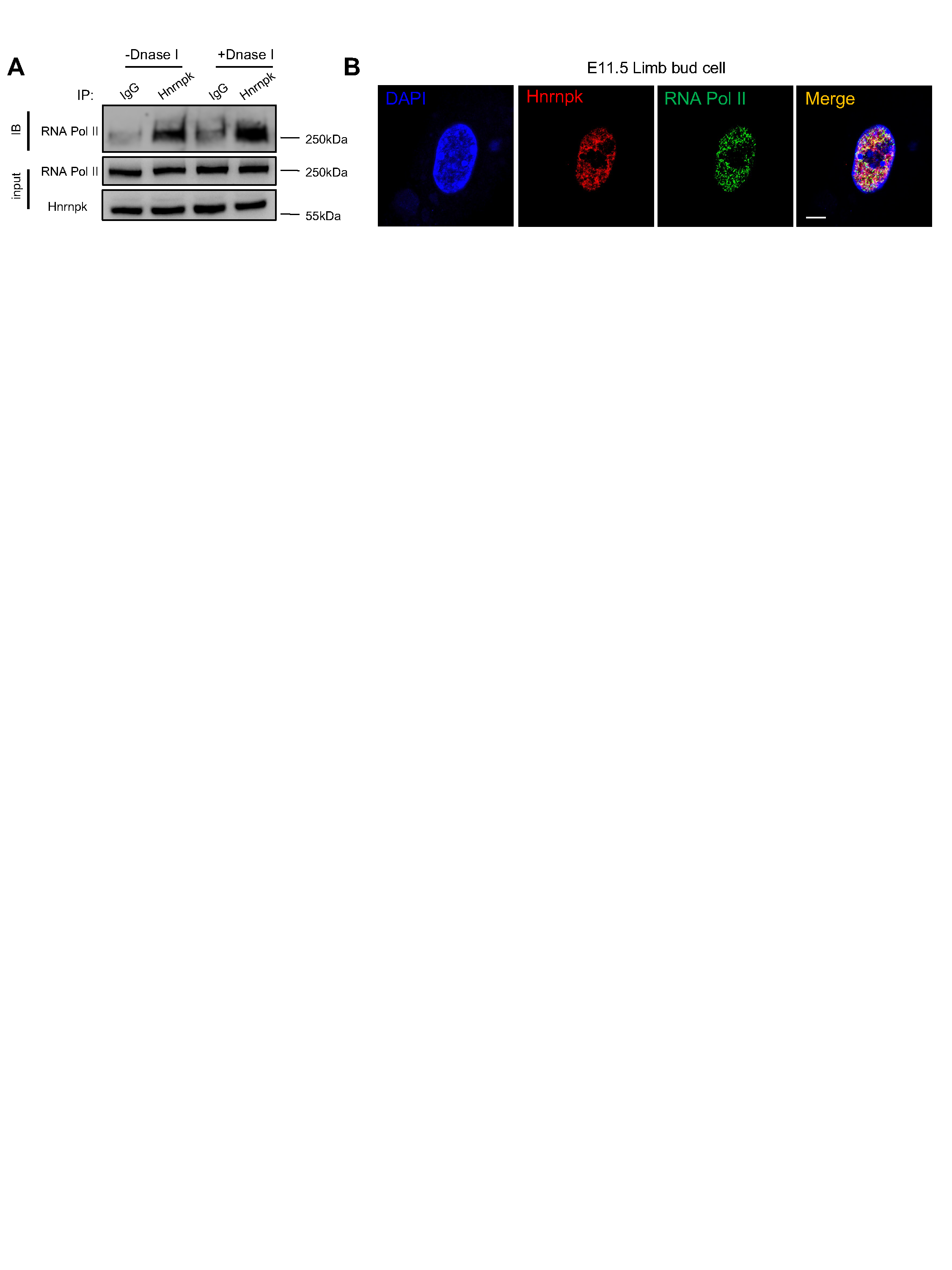
Fig. S10. Hnrnpk** **interacts with RPII in limb bud cells.**

A. IP assay between Hnrnpk and RPII at E11.5 in WT limb bud after treatment with PBS or DNase I.

B. Subcellular location of Hnrnpk and RPII at E11.5 in primary WT limb bud cell. Scale bar: 10 μm.

**Table S1.** Primer sequences for qPCR.

| Sequence for qPCR | | |
| --- | --- | --- |
| Gene | Direction | Sequence (5'-3') |
| *Actb* | forward | GGCTGTATTCCCCTCCATCG |
|  | reversed | CCAGTTGGTAACAATGCCATGT |
| *Hnrnpk* | forward | CCGTACAGACTACAATGCCAG |
|  | reversed | GCCCTCTTCCAAGGTAGGGAT |
| *Shh* | forward | AAAGCTGACCCCTTTAGCCTA |
|  | reversed | TTCGGAGTTTCTTGTGATCTTCC |
| *Fgf8* | forward | AGGGGAAGCTAATTGCCAAGA |
|  | reversed | CCTTGCGGGTAAAGGCCAT |
| *Lmx1b* | forward | TTCCTGATGCGAGTCAACGAG |
|  | reversed | TCCGATCCCGGAAGTAGCAG |
| *Fgf10* | forward | TTTGGTGTCTTCGTTCCCTGT |
|  | reversed | TAGCTCCGCACATGCCTTC |
| *HoxD9* | forward | GCACCCTCAGCAACTACTACG |
|  | reversed | AAAACTACACGAGGCGAACTC |
| *HoxD10* | forward | ACCTATGGAATGCAAACCTGTG |
|  | reversed | TCTGTCCAACTGTCTACTTGAGG |
| *HoxD11* | forward | AAAAGACTCCAACTCTCTCGGA |
|  | reversed | AGACGGTCCCTGTTCAGTTTC |
| *HoxD12* | forward | CTATGTGGGCTCGCTTCTGAA |
|  | reversed | GGCTCTCAGGTTGGAAAAGTAG |
| *HoxD13* | forward | TACCACTTCGGCAACGGTTAC |
|  | reversed | CCCGACACGTCCATGTACTTC |
| *Trp53* | forward | CTCTCCCCCGCAAAAGAAAAA |
|  | reversed | CGGAACATCTCGAAGCGTTTA |
| *Cdkn1a* | forward | CCTGGTGATGTCCGACCTG |
|  | reversed | CCATGAGCGCATCGCAATC |
| *Bax* | forward | TGAAGACAGGGGCCTTTTTG |
|  | reversed | AATTCGCCGGAGACACTCG |

**Table S2.** DNA sequences for constructing WISH probes from cDNA.

| Sequence for WISH | | |
| --- | --- | --- |
| Gene | Direction | Sequence (5'-3') |
| *Lmx1b* | forward | TGAAGAGTGAGGATGAAGATGG |
|  | reversed-1 | GAGTCTGAGCGGGTGGTG |
|  | reversed-2 | CAGTGAATTGTAATACGACTCACTATAGGGAGA GAGTCTGAGCGGGTGGTG |
| *Fgf10* | forward | CTTCCAGTATGTTCCTTCTGATGAGAC |
|  | reversed-1 | GTACGGACAGTCTTCTTCTTGGTCCC |
|  | reversed-2 | CAGTGAATTGTAATACGACTCACTATAGGGAGA GTACGGACAGTCTTCTTCTTGGTCCC |
| *Sox9* | forward | CCACCCACCACTCCC |
|  | reversed-1 | GATGCCGTAACTGCC |
|  | reversed-2 | CAGTGAATTGTAATACGACTCACTATAGGGAGA GATGCCGTAACTGCC |
| *Col2a1* | forward | GGCTGCCGGGTCTCCTGCC |
|  | reversed-1 | TGGGCACCACCAGCC |
|  | reversed-2 | CAGTGAATTGTAATACGACTCACTATAGGGAGA TGGGCACCACCAGCC |
| *Hnrnpk* | forward-1 | TTCCCCAACACCGAAACCAA |
|  | reversed-1 | CCATGCCATCATAGCGGTCT |
|  | forward-2 | CAGTGAATTGAATTAACCCTCACTAAAGGGGAGA TTCCCCAACACCGAAACCAA |
|  | reversed-2 | CAGTGAATTGTAATACGACTCACTATAGGGGAGA CCATGCCATCATAGCGGTCT |

**Table S3.** DNA sequences of sgRNA.

| sgRNA for dCas9 | | | | |
| --- | --- | --- | --- | --- |
| Gene | | PAM | | Sequence |
| *Shh-sgRNA* | | AGG | | AGGGAGAGCCGAGCGCAAGG |
| *Fgf10-sgRNA* | | AGG | | GAAGGGTAAGACCTGCTGCG |
| *Lmx1b-sgRNA* | | TGG | | GGCGACGGCACTATTTGACG |
|  |  | |  | |
