# Supplementary figures and images for: Hnrnpk is essential for embryonic limb bud development as a transcription activator and a collaborator of insulator protein Ctcf
Source: Cell Death Differ. 2023 Aug 22;30(10):2293–308. doi: 10.1038/s41418-023-01207-z (PMC10589297; doi:10.1038/s41418-023-01207-z)

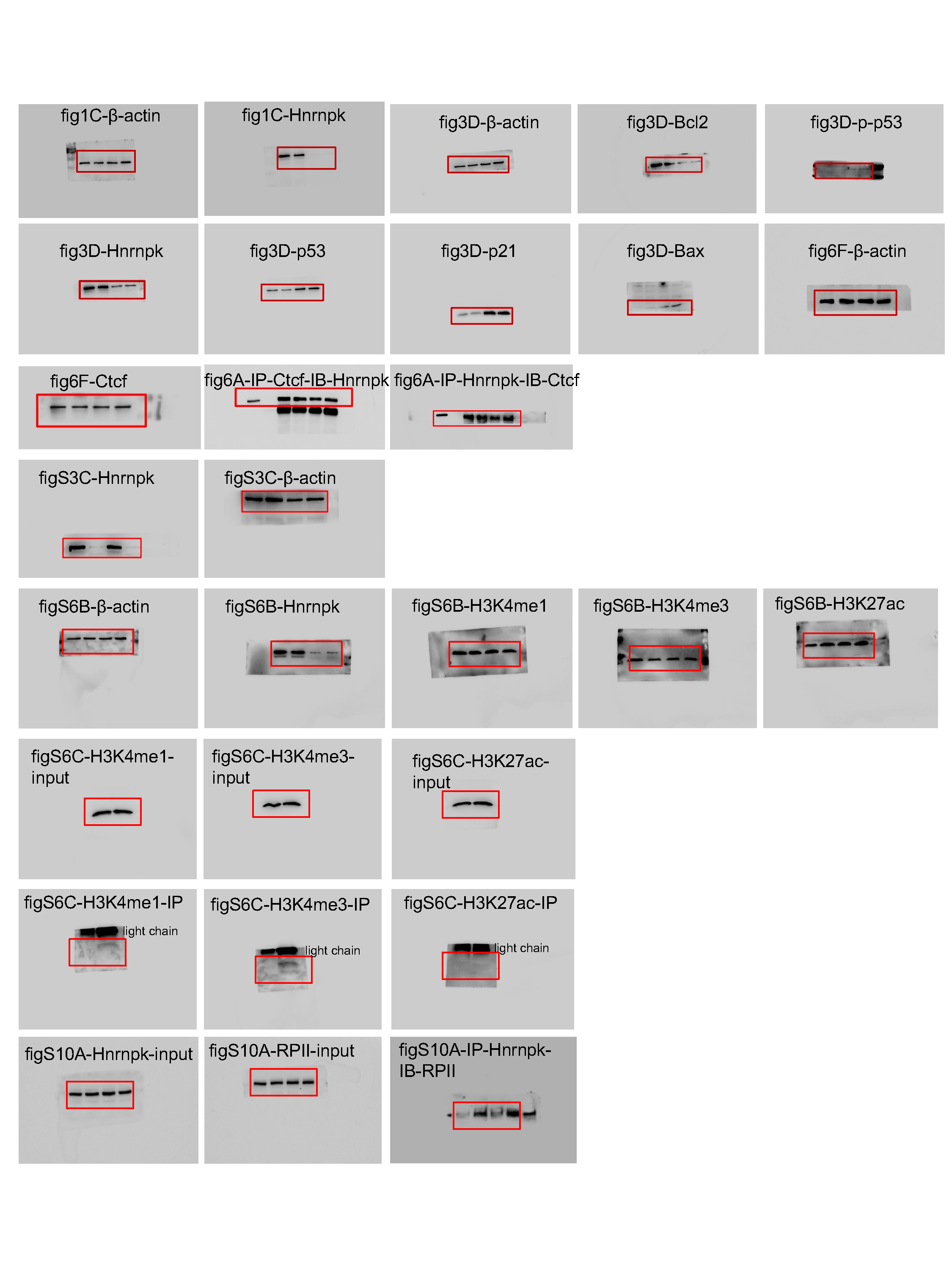

Supplement: Supplementary file 2 — Supplementary materials Western blot [file 41418_2023_1207_MOESM2_ESM.tif]
